# The HUNT study identifies host genetic factors reproducibly associated with human gut microbiota composition

**DOI:** 10.1038/s41588-026-02502-4

**Published:** 2026-02-13

**Authors:** Marta Riise Moksnes, Eivind Coward, Maria Nethander, Koen Dekkers, Louise Grahnemo, Anna E. Törnqvist, Lei Li, Per Lundmark, Kamalita Pertiwi, Gabriel Baldanzi, Robin Mjelle, Janne Marie Moll, Aron Charles Eklund, Henrik Bjørn Nielsen, Johan Svensson, Arnulf Langhammer, Guro F. Giskeødegård, Ben Brumpton, Rebecka Hjort, Eivind Ness-Jensen, Gunnar Engström, Thaher Pelaseyed, Karl Michaëlsson, Marju Orho-Melander, Tove Fall, Kristian Hveem, Claes Ohlsson

**Affiliations:** 1 Department of Internal Medicine and Clinical Nutrition, Institute of Medicine, Sahlgrenska Osteoporosis Centre, Centre for Bone and Arthritis Research at the Sahlgrenska Academy, University of Gothenburg, Gothenburg, Sweden; 2 HUNT Center for Molecular and Clinical Epidemiology, Department of Public Health and Nursing, Norwegian University of Science and Technology, Trondheim, Norway; 3 Molecular Epidemiology, Department of Medical Sciences, Uppsala University, Uppsala, Sweden; 4 Department of Cancer and Molecular Medicine, Norwegian University of Science and Technology, Trondheim, Norway; 5 Department of Pathology, St. Olavs Hospital, Trondheim, Norway; 6 Clinical Microbiomics A/S, Copenhagen, Denmark; 7 Region Västra Götaland, Skaraborg Central Hospital, Department of Internal Medicine, Skövde, Sweden; 8 HUNT Research Centre, Department of Public Health and Nursing, NTNU, Norwegian University of Science and Technology, Levanger, Norway; 9 Department of Medicine, Levanger Hospital, Nord-Trøndelag Hospital Trust, Levanger, Norway; 10 Department of Surgery, St. Olavs University Hospital, Trondheim, Norway; 11 Clinic of Medicine, St. Olavs Hospital, Trondheim University Hospital, Trondheim, Norway; 12 Department of Molecular Medicine and Surgery, Karolinska Institutet, Karolinska University Hospital, Stockholm, Sweden; 13 Department of Clinical Sciences in Malmö, Lund University, Malmö, Sweden; 14 Department of Medical Biochemistry and Cell Biology, Institute of Biomedicine, University of Gothenburg, Gothenburg, Sweden; 15 Medical Epidemiology, Department of Surgical Sciences, Uppsala University, Uppsala, Sweden; 16 Region Västra Götaland, Sahlgrenska University Hospital, Department of Drug Treatment, Gothenburg, Sweden

**Keywords:** Genome-wide association studies, Epidemiology, Bacteriology

## Abstract

The gut microbiota is associated with human health and disease. Here we conducted a genome-wide association study of host genetic factors influencing gut microbiota composition in 12,652 individuals from the Trøndelag Health Study (HUNT), with replication in Nordic cohorts (*n* = 16,017–21,976). We identified 12 reproducible SNP–species associations across six genomic loci, including known (*LCT*, *ABO*) and novel (*HLA-DQB1*, *MUC12*, *SLC37A2*, *FUT2*) regions. Additionally, we detected genetic signals associated with gut microbiota functional modules at three loci (*LCT*, *ABO*, *FUT2*). Follow-up analyses suggest that these host–microbiota associations are linked to the pathogenesis of celiac disease and hemorrhoidal disease. Mendelian randomization analyses provided evidence supporting a causal effect of body mass index on gut microbiota composition. These findings highlight the interplay between host genetics and gut microbiota for human health and disease.

## Abstract (web summary)

Genome-wide analyses in the Norwegian HUNT study with replication in Swedish and Finnish cohorts identify host genetic variants associated with gut microbiome features and provide evidence of a causal effect of body mass index on gut microbiota composition.

## Main

The human gut microbial community is highly diverse and plays an important role in normal gut physiology, including digestion, metabolism and immune regulation^1^. The gut microbiota has also been associated with a range of diseases, but most causal effects of the microbiota on human health are still to be established^2–4^. The composition and function of the gut microbiota are influenced by multiple factors, including diet, medication, age and host genetics^5^.

Twin studies have demonstrated that the gut microbiota composition is influenced by host genetics^6,7^, but previous genome-wide association studies (GWASs) have identified only two genetic loci (the *LCT* and *ABO* loci) reproducibly associated with gut microbiota composition^2–4,8^. Earlier studies have been underpowered, lacked replication and/or relied on 16S ribosomal RNA gene sequencing profiles with low taxonomic coverage^2–4,9–11^. To our knowledge, no previous GWAS has identified a replicated host genetic signal for gut microbiota functionality.

Several host traits and lifestyle factors have been associated with human gut microbial composition, including body mass index (BMI), physical activity, smoking and different diseases^12^. However, observational associations may be biased by confounding, and the causal direction for a major factor such as BMI remains unclear^12,13^. Mendelian randomization (MR) methods have been developed to infer causal relationships from genetic data, and are, under certain assumptions, less influenced by confounding and reverse causality than traditional observational designs. Nevertheless, using MR to evaluate bidirectional causal relationships between gut microbiota composition and host traits requires robust genetic instruments for gut microbial exposures and well-powered GWAS summary statistics for gut microbial outcomes. Unfortunately, previous host GWASs of gut microbiota composition using metagenome sequence data have generally been underpowered (*n* ≤ 7,738) as sources for exposure and outcome data in MR^2–4,9–11^.

**Fig. 1:**
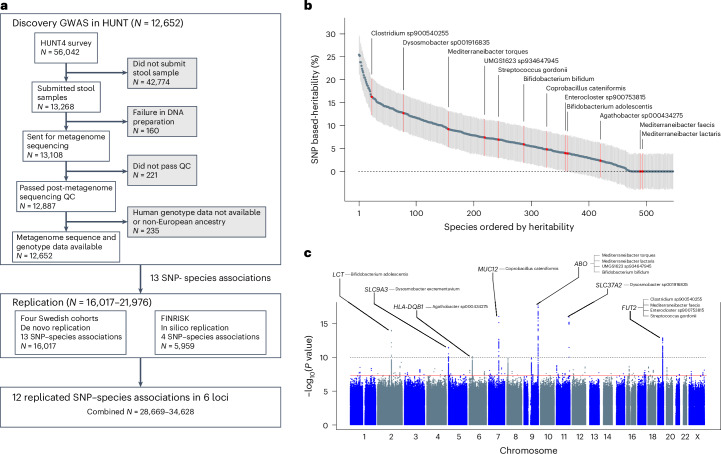
GWAS and replications of gut microbiota species relative abundance. **a**, Overall design of the discovery GWAS in HUNT and the subsequent replications in four Swedish cohorts (all 13 SNP–species associations for replication) and in the FINRISK cohort (four SNP–species associations available for replication). **b**, Total SNP-based heritability, estimated as the ratio of genetic variance (V_g_) to phenotypic variance (V_p_), given as mean with 95% confidence intervals for the 546 evaluated species using genome-wide complex trait analysis (GCTA). **c**, Manhattan plot summarizing the SNP associations with the 546 gut microbiota species evaluated in the discovery GWAS. Results after replication are shown in Table 1 and Supplementary Table 7. The unadjusted *P* values are based on two-sided *z*-tests. Red line indicates genome-wide significance (*P* < 5.0 × 10^−8^). Gray dotted line indicates study-wide significance threshold adjusted for number of effective tests (*P* < 1.3 × 10^−10^). The identified genetic loci (index SNP ± 500 kb) are annotated with the species with study-wide significant associations. QC, quality control.

To unravel host genetic factors influencing gut microbiota composition, we performed a large-scale GWAS including 12,652 participants with metagenome sequence data available in the Trøndelag Health Study (HUNT). The results were replicated in large Nordic cohorts (*n* = 16,017–21,976; Fig. 1a). To further understand the host-microbiota interactions, we performed a GWAS of gut microbiota functional potential (Kyoto Encyclopedia of Genes and Genomes (KEGG) modules) in 12,652 participants in HUNT, followed by replication in four Swedish cohorts (*n* = 16,017; Supplementary Fig. 4a). Our subsequent disease-focused phenome-wide association study (PheWAS) linked the novel findings in the gut microbiota GWAS to plausible diseases. Finally, taking advantage of the summary statistics from our large GWAS in the discovery cohort, we explored possible causal associations between BMI and gut microbiota composition.

## Results

### GWAS of gut microbiota species

**Fig. 2:**
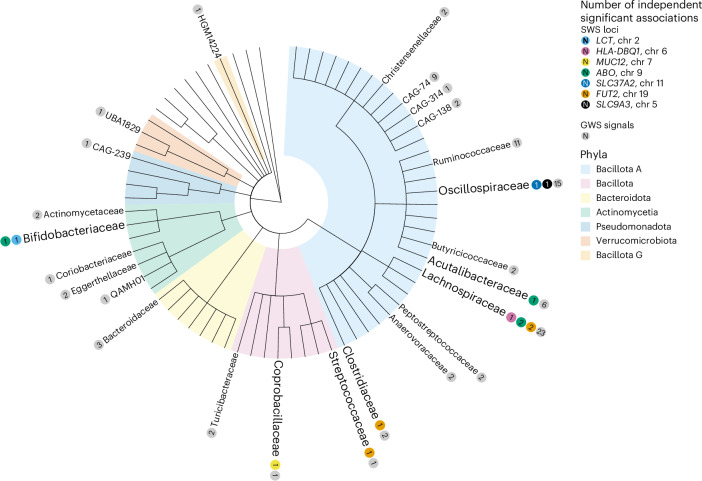
Cladogram of the gut microbiota associations. Overview of the gut microbiota species for families with independent genetic signals associated with species. The numbers of independent significant association signals are given within the circles either for those that were study-wide significant (SWS; *P* < 1.3 × 10^−10^), given per significant locus (one color per locus) or for all genome-wide significant associations not including the SWS associations (GWS; 1.3 × 10^−10^ ≤ *P* < 5.0 ^×^ 10^−8^, given in gray). The total number of independent SWS signals selected for replication are 13, located in 7 different loci. The total number of GWS signals, also including the SWS signals, is 106. For further details of individual associations, see Table 1 and Supplementary Table 6.

We evaluated associations between the relative abundance of 546 gut microbiota species (prevalence ≥30 %) and 7,971,622 genetic variants (minor allele frequency (MAF) ≥ 0.01) in 12,652 HUNT participants (Fig. 1, Supplementary Tables 1 and 2 and Supplementary Note). We identified genetic signals in seven loci, including 13 SNP-species signals (*P* < 1.3 × 10^−10^; Fig. 1c, Table 1 and Supplementary Fig. 3) selected for replication. Among the 13 selected SNP–species associations, 12 were successfully replicated (concordant direction of effect in the replication data and *P* < 3.8 × 10^−3^ (Bonferroni correction for 13 comparisons); *n* = 16,017–21,966; Table 1, Figs. 1a and 2 and Supplementary Fig. 3).

**Table 1:** Replicated GWAS signals associated with gut microbiota species

Chr	BP (hg19)	RSID	REF	EA	Locus			Discovery				Replication				Meta-analysis				Novel locus
						Outcome species		HUNT (*n* = 12,652)				Swedish cohorts (*n* = 16,017)				(*n* = 28,669)				
						Name	Prevalence	EAF	Beta	s.e.	*P*value	EAF	Beta	s.e.	*P*value	EAF	Beta	s.e.	*P*value	
**Replicated signals**																				
**2**	**136616754**	rs182549	**C**	**T**	***LCT***	***Bifidobacterium adolescentis***	**97%**	0.76	−0.14	0.02	1.3 × 10^−14^	0.72	−0.10	0.01	8.5 × 10^−17^	0.73	−0.11	0.01	3.8 × 10^−29^	No
**6**	**32626348**	rs28407950	**C**	**T**	***HLA-DQB1***	***Agathobacter sp000434275***	**65%**	0.27	0.09	0.01	9.8 × 10^−11^	0.29	0.05	0.01	1.2 × 10^−7^	0.28	0.06	0.01	6.3 × 10^−16^	Yes
**7**	**100632790**	rs4556017	**C**	**T**	***MUC12***	***Coprobacillus cateniformis***	**33%**	0.84	0.11	0.01	1.1 × 10^−16^	0.83	0.10	0.01	6.0 × 10^−17^	0.83	0.10	0.01	5.1 × 10^−32^	Yes
9	136142217	rs644234	T	G	*ABO*	*Mediterraneibacter lactaris*	73%	0.38	0.09	0.01	9.1 × 10^−14^	0.41	0.05	0.01	2.3 × 10^−7^	0.40	0.07	0.01	2.3 × 10^−18^	No
**9**	**136146597**	rs550057	**C**	**T**	*ABO*	***Mediterraneibacter torques***	**90%**	0.30	0.11	0.01	2.4 × 10^−18^	0.31	0.10	0.01	1.8 × 10^−18^	0.31	0.08	0.01	8.3 × 10^−25^	
9	136146597	rs550057	C	T	*ABO*	*UMGS1623 sp934647945*	73%	0.30	0.11	0.01	1.9 × 10^−17^	0.31	0.07	0.01	3.7 × 10^−10^	0.31	0.11	0.01	5.4 × 10^−35^	
9	136149229	rs505922	T	C	*ABO*	*Bifidobacterium bifidum*	51%	0.36	−0.09	0.01	7.7 × 10^−15^	0.40	−0.04	0.01	2.2 × 10^−7^	0.39	−0.06	0.01	1.5 × 10^−18^	
**11**	**124942269**	rs73024305	**G**	**C**	***SLC37A2***	***Dysosmobacter sp001916835***	**91%**	0.08	0.19	0.02	1.4 × 10^−16^	0.06	0.07	0.02	9.5 × 10^−4^	0.07	0.13	0.02	1.2 × 10^−15^	Yes
19	49203829	rs35106244	C	T	*FUT2*	*Mediterraneibacter faecis*	93%	0.40	−0.08	0.01	5.0 × 10^−11^	0.40	−0.06	0.01	6.0 × 10^−8^	0.40	−0.07	0.01	6.0 × 10^−17^	No*
19	49215095	rs4002471	C	T	*FUT2*	*Streptococcus gordonii*	56%	0.48	0.07	0.01	8.3 × 10^−11^	0.49	0.04	0.01	4.5 × 10^−5^	0.49	0.05	0.01	2.4 × 10^−13^	
19	49218060	rs35866622	C	T	*FUT2*	*Enterocloster sp900753815*	34%	0.43	0.07	0.01	5.9 × 10^−12^	0.43	0.05	0.01	8.8 × 10^−9^	0.43	0.06	0.01	9.8 × 10^−19^	
**19**	**49228272**	rs2287921	**T**	**C**	*FUT2*	***Clostridium sp900540255***	**31%**	0.48	−0.07	0.01	1.7 × 10^−13^	0.49	−0.06	0.01	1.1 × 10^−9^	0.49	−0.06	0.01	2.3 × 10^−21^	
**Signal not replicated**																				
5	528609	rs6880820	T	C	*SLC9A3*	*Dysosmobacter excrementavium*	40%	0.18	0.09	0.01	4.3 × 10^−12^	0.19	0.02	0.01	7.4 × 10^−2^	0.19	0.05	0.01	1.1 × 10^−8^	Not replicated

Locus values were assigned by the Open Target Platform. Bold indicates the most significant SNP–species association in a locus. The prevalences for the outcome species are given for HUNT. Beta values in HUNT and the Swedish cohorts are given as standard deviation change of inverse rank transformed species per effect allele. *P* value represents the unadjusted *P* value from a two-sided *z*-test. Novel locus indicates a locus not previously shown to be reproducibly associated with any microbial species. BP, base pair; Chr, chromosome; EA, effect allele; EAF, effect allele frequency; REF, reference (non-effect) allele; s.e., standard error.

*The *FUT2* locus has been reported to pass the traditional genome-wide significance level (*P* < 5.0 × 10^−8^), but with no replication and not reaching study-wide significance in previous studies.

The replicated SNP–species associations included two well-known gut-microbiota-associated loci (*LCT* on Chr2 and *ABO* on Chr9)^2–4,8^. In addition, the identified *FUT2* locus has previously been reported to pass the traditional genome-wide significance level (*P* < 5.0 × 10^−8^), but with no replication^2^. Further, we identified replicated genetic signals in three loci (*HLA-DQB1* on Chr6, *MUC12* on Chr7 and *SLC37A2* on chr 11) not previously associated with the relative abundance of gut microbiota species in any GWAS (Table 1).

We performed several sensitivity analyses (excluding participants on antibiotic treatment, adjusting for bowel motility, only including unrelated individuals, using the centered log-ratio transformation, excluding cohabitation), revealing essentially unchanged effect estimates for the SNP-species associations (Supplementary Tables 8 and 9 and Supplementary Note).

GWAS on α-diversity parameters identified one genome-wide significant genetic signal for Shannon diversity index (rs12140644-G; beta = −0.11, standard error (s.e.) = 0.02, *P* = 2.6 × 10^−8^), whereas no significant signal was observed for richness in HUNT.

GCTA revealed SNP heritability estimates (*h*^2^) of between 0% and 25% for the 546 evaluated species (mean heritability of 6.8%; Fig. 1b and Supplementary Table 3). The heritability was higher for species with genome-wide significant genetic associations compared to those without (Supplementary Table 4). The SNP heritability for the two α-diversity measures, Shannon diversity index and richness, was 15.6 ± 4.1% and 24.5 ± 4.2%, respectively (Supplementary Note).

### GWAS of KEGG functionality modules

To explore the biology underlying host genetics-gut microbiota relationships, we performed GWAS on 461 gut microbiota KEGG functionality modules (prevalence ≥ 30%; Supplementary Fig. 4a and Supplementary Table 24) in HUNT. We identified genetic signals in four loci, including eight SNP-KEGG functionality module associations (*P* < 4.9 × 10^−10^; Table 2, Supplementary Fig. 4a,c), which were selected for replication. Among these, six SNP-KEGG functionality module associations were successfully replicated (concordant direction of effect in the replication data and *P* < 6.25 × 10^−3^ (Bonferroni correction for eight comparisons)) in the Swedish replication data sets (*n* = 16,017; Table 2 and Supplementary Fig. 4a), none of which have been previously reported (Supplementary Note). The replicated genetic signals for KEGG functionality modules were found at three loci (*LCT*, *ABO* and *FUT2*) (Table 2). The identified genetic signals for the KEGG functionality modules were either the same SNP or a SNP strongly linked to the SNP identified for the relative abundance of gut microbiota species at the corresponding loci (Tables 1 and 2 and Supplementary Fig. 3g–i). Using GCTA, SNP heritability estimates for KEGG functionality modules varied between 0% and 26% (mean heritability of 7.2%; Supplementary Fig. 4b and Supplementary Table 10).

**Table 2:** Replicated GWAS signals associated with KEGG functionality modules

Chr	BP (hg19)	RSID	Module	Name	REF	EA	Locus	Discovery				Replication				Meta-analyses			
								HUNT (*n* = 12,652)				Swedish cohorts (*n* = 16,017)				Discovery + Replication (*n* = 28,669)			
								EAF	Beta	s.e.	*P* value	EAF	Beta	s.e.	*P* value	EAF	Beta	s.e.	*P* value
**Replicated signals**																			
2	136608646	rs4988235	M00443	SenX3-RegX3 (phosphate starvation response) two-component regulatory system	G	A	*LCT*	0.76	−0.16	0.02	2.6 × 10^−18^	0.72	−0.12	0.01	4.8 × 10^−21^	0.73	−0.13	0.01	5.5 × 10^−37^
2	136608646	rs4988235	M00233	Glutamate transport system	G	A	*LCT*	0.76	−0.16	0.02	3.1 × 10^−18^	0.72	−0.12	0.01	1.4 × 10^−21^	0.73	−0.13	0.01	2.7 × 10^−37^
2	136616754	rs182549	M00244	Putative zinc/manganese transport system	C	T	*LCT*	0.76	−0.15	0.02	4.0 × 10^−15^	0.72	−0.12	0.01	3.3 × 10^−20^	0.73	−0.13	0.01	3.3 × 10^−33^
2	136616754	rs182549	M00168	CAM (crassulacean acid metabolism), dark	C	T	*LCT*	0.76	−0.13	0.02	2.7 × 10^−11^	0.72	−0.09	0.01	3.8 × 10^−13^	0.73	−0.10	0.01	2.0 × 10^−22^
9	136155000	rs635634	M00228	Putative glutamine transport system	C	T	*ABO*	0.21	0.10	0.02	1.3 × 10^−10^	0.22	0.05	0.01	6.8 × 10^−5^	0.22	0.07	0.01	6.3 × 10^−13^
19	49218060	rs35866622	M00719	Ihk-Irr (virulence regulation) two-component regulatory system	C	T	*FUT2*	0.43	0.07	0.01	1.2 × 10^−10^	0.43	0.03	0.01	2.0 × 10^−3^	0.43	0.05	0.01	6.1 × 10^−11^
**Signals not replicated**																			
8	11630429	rs804264	M00518	GlnK-GlnL (glutamine utilization) two-component regulatory system	C	A	*NEIL2*	0.44	−0.08	0.01	6.1 × 10^−11^	0.44	−0.02	0.01	2.2 × 10^−2^	0.44	−0.05	0.01	2.1 × 10^−9^
8	11625235	rs804273	M00747	Bacitracin transport system	G	C	*NEIL2*	0.52	−0.08	0.01	1.0 × 10^−10^	0.53	−0.03	0.01	8.2 × 10^−3^	0.52	−0.05	0.01	5.0 × 10^−10^

BP, base pair; Chr, chromosome; EA, effect allele; EAF, effect allele frequency; REF, reference (non-effect) allele. Beta values are given as standard deviation of inverse rank-transformed KEGG functionality module per effect allele. *P* value represent unadjusted *P* value from a two-sided *z*-test

### Genetic signal at the *LCT* locus

We observed that the index SNP rs182549-T allele in *LCT*, strongly linked with the lactase persistence rs4988235-A allele (linkage disequilibrium (LD) correlation *r*^2^ = 0.996 in the European ancestry 1000 Genomes reference panel), was reproducibly associated with lower relative abundance of *Bifidobacterium adolescentis* (*P* = 2.1 × 10^−45^; Table 1 and Supplementary Fig. 3a) and decreased functional potential of four KEGG functionality modules including SenX3-RegX3 (phosphate starvation response) two-component regulatory system (*P* = 5.5 × 10^−37^), glutamate transport system (*P* = 2.7 × 10^−37^), putative zinc/manganese transport system (*P* = 3.3 × 10^−33^), and crassulacean acid metabolism (*P* = 2.0 × 10^−22^; Table 2). The relative abundance of *Bifidobacterium adolescentis* was strongly correlated with all four identified KEGG functionality modules, especially with the SenX3-RegX3 (phosphate starvation response) two-component regulatory system (60% of the variance explained) and the glutamate transport system (61% of the variance explained; Supplementary Table 35).

### Genetic signal at the *HLA-DQB1* locus

The *Agathobacter sp0004342*75 index SNP (rs28407950, *P* = 4.0 × 10^−20^; Supplementary Table 7) was assigned by the Open Targets platform^14^ to have functional implications for the nearby *HLA-DQB1* gene, with expression quantitative trait loci (eQTLs) in the small intestine and in sigmoid colon (Supplementary Tables 11 and 12). Our disease-focused PheWAS of rs28407950 revealed strong associations for rs28407950-T, which is associated with higher relative abundance of *Agathobacter sp0004342*75 and reduced risk of major autoimmune diseases such as type 1 diabetes (*P* = 1.2 × 10^−183^), asthma (*P* = 4.3 × 10^−118^) and celiac disease (*P* = 2.1 × 10^−84^; Supplementary Table 13).

Observationally, the relative abundance of *Agathobacter sp0004342*75 was inversely associated with the plausible gastrointestinal condition celiac disease in HUNT (*n* = 240 cases and 12,437 controls; odds ratio = 0.53, 95% confidence interval 0.44-0.63 per standard deviation (s.d.) increase in relative abundance of *Agathobacter sp0004342*75, *P* = 3.7 × 10^−12^). This association is also illustrated in a distribution plot (Supplementary Fig. 6a), and the mean relative abundance of *Agathobacter sp0004342*75 was 0.046% in celiac disease cases and 0.120% in controls (Supplementary Note).

MR revealed that having celiac disease was causally associated with reduced relative abundance of *Agathobacter sp000434275* (beta = −0.036, s.e. = 0.014, *P* = 9.9 × 10^−3^; Supplementary Tables 25 and 26), whereas the possible impact of *Agathobacter sp000434275* on celiac disease is unclear (Supplementary Note).

### Genetic signal at the *MUC12* locus

**Fig. 3:**
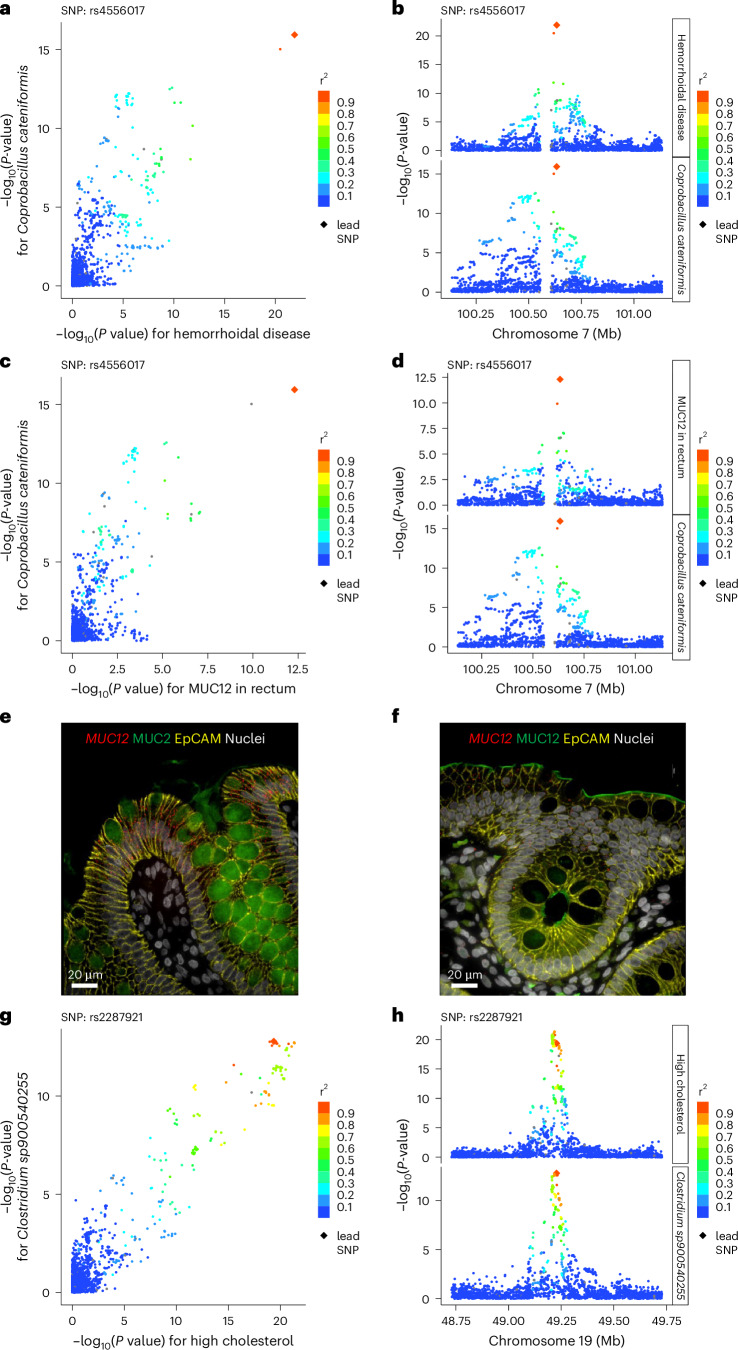
Follow-up analyses of some novel genetic signals with possible implications for associated diseases. **a**,**b**, Bayesian colocalization analyses demonstrating colocalized genetic signals for *C. cateniformis* and hemorrhoidal disease, with a posterior probability of a single shared causal variant of 99.8%. **c**,**d**, Bayesian colocalization analyses demonstrating colocalized genetic signals for *C. cateniformis* and *MUC12* expression in rectum, with a posterior probability of a single shared causal variant of 99.96%. **e**,**f**, *MUC12* expression in human sigmoid colon. **e**, *MUC12* mRNA is indicated in red, MUC2 protein as a marker of Goblet cells in green, EpCAM (epithelial cell adhesion molecule) protein in yellow as a marker of colonocytes and nuclei in white. **f**, *MUC12* mRNA is indicated in red, MUC12 protein in green, EpCAM protein in yellow as a marker of colonocytes and nuclei in white. The experiment was repeated three times using samples from four different individuals. **g**,**h**, Bayesian colocalization analyses providing evidence for colocalization of the genetic signals for the relative abundance of *Clostridium sp900540255* and high cholesterol, with a posterior probability of a single shared causal variant of 80.3%.

The Open Targets platform assigned the *Coprobacillus cateniformis* index SNP (rs4556017, *P* = 1.1 × 10^−37^; Supplementary Table 7) to have functional implications for the nearby *MUC12* gene (Supplementary Table 14). For rs4556017, there is an eQTL for *MUC12* in the rectum (Supplementary Table 15). Colocalization analyses provided strong evidence (posterior probability, PP4 = 99.96%) for a shared causal variant in the *MUC12* locus affecting both the relative abundance of *C. cateniformis* and the expression of *MUC12* in rectum (Fig. 3c,d). The rs4556017-T allele was associated with both increased relative abundance of *C. cateniformis* and increased *MUC12* expression in rectum, compared with the reference C allele.

Our disease-focused PheWAS of the top associated SNP rs4556017 revealed a robust association with hemorrhoidal disease (*P* = 1.3 × 10^−22^; Supplementary Table 16). Using Bayesian colocalization analyses, we found strong evidence (PP4 = 99.8%) for a shared causal variant in the *MUC12* locus affecting both the relative abundance of *C. cateniformis* and the risk of hemorrhoidal disease (Fig. 3a,b and Supplementary Note).

We observed that the *MUC12* transcript displayed the highest expression in colon among all 54 investigated human tissues available in the Genotype-Tissue Expression (GTEx) portal (Supplementary Fig. 7). To determine the cellular distribution of *MUC12* expression in the human sigmoid colon, we performed in situ hybridization (RNAscope). *MUC12* mRNA was abundantly expressed in colonocytes, but expression was also observed in MUC2-positive goblet cells (Fig. 3e,f), whereas MUC12 protein was observed at the luminal surface of colonocytes (Fig. 3f and Supplementary Fig. 8).

### Genetic signal at the *SLC37A2* locus

We observed a novel genetic signal (index SNP rs73024305) for the relative abundance of *Dysosmobacter sp001916835* in the *SLC37A2* (solute carrier family 37 (glycerol-3-phosphate transporter) member 2) locus at Chr11 (Table 1, Supplementary Fig. 3e and Supplementary Tables 17 and 18). Evidence from the Open Targets platform for *SLC37A2* being the underlying gene for this genetic signal includes correlation between rs73024305 and the transcriptional activity of enhancers and transcription start sites of *SLC37A2* using the FANTOM5 expression atlas^15^ (Supplementary Table 17) and an eQTL (Supplementary Table 18). The PheWAS of the top associated SNP rs73024305 did not reveal robust associations with diseases (Supplementary Table 19). Association analyses with circulating metabolites demonstrated that *Dysosmobacter sp001916835* was associated with reduced levels of the secondary bile acid isoursodeoxycholate (*P* = 5.1 × 10^−160^, *r*_*s*_ = −0.29) and increased levels of the metabolite 3-phenylpropionate (*P* = 1.3 × 10^−161^, *r*_*s*_ = 0.29; Supplementary Table 27).

### Interaction between genetic variants at *ABO* and *FUT2* loci

The strongest replicated association in the *FUT2* locus was observed between rs2287921 and *Clostridium sp900540255* (*P* = 3.4 × 10^−21^; Fig. 1c and Table 1). This SNP is in LD with the functional variant rs601338 (*r*^2^ = 0.65; *D*′ = 0.87 in European-ancestry populations, A/A = non-secretor for rs601338 is associated with C/C for rs2287921) that introduces a stop codon in *FUT2* (Supplementary Tables 20 and 21)^16^. The association between the secretor SNP rs601338 and *Clostridium sp900540255* was also significant in HUNT (*P* = 8.8 × 10^−13^). The other three gut microbiota species index SNPs in this locus were also strongly linked with the functional *FUT2* variant rs601338 (Table 1, Supplementary Fig. 3f and Supplementary Table 28).

*FUT2* encodes the enzyme alpha-1,2-fucosyltransferase 2, required for synthesizing fucosylated mucin glycans in the intestinal mucosa^16^. As alpha-1,2-fucosyltransferase 2 is required for the expression of *ABO* antigens on the intestinal mucosa, we hypothesized that there might be an interaction between the top genetic signal identified in the *ABO* locus (rs550057) and the top genetic signal in the *FUT2* locus (rs2287921). We observed a strong interaction between these two SNPs (*P* = 5.2 × 10^−7^ for the SNP × SNP interaction term) for the association with the relative abundance of *Mediterraneibacter torques* (Supplementary Table 29 and Supplementary Note).

Our disease-focused PheWAS of the index SNP rs2287921 in the *FUT2* locus revealed an association with a composite cardiovascular-related outcome parameter (*P* = 6.9 × 10^−18^; Supplementary Table 22). The rs2287921-T allele (responsible for the formation of the secretor status) was associated with a decreased risk of cardiovascular-related outcomes. Separate analyses revealed that the observed association was mainly driven by the strong association of rs2287921-T with a reduced risk of high cholesterol and hypertension (Supplementary Tables 22 and 34). Bayesian colocalization analyses provided strong evidence for a shared causal variant in the *FUT2* locus for relative abundance of *Clostridium sp900540255* and high cholesterol (PP4 = 80%; Fig. 3g,h and Supplementary Note).

Analyses of circulating metabolites known to be associated with poor cardiometabolic health revealed that *Mediterraneibacter faecis*, linked to FUT2 secretion status, was associated with reduced levels of the metabolites p-cresol sulphate (*P* = 1.0 × 10^−26^, *r*_*s*_ = −0.12) and phenylacetate (*P* = 4.6 × 10^−33^, *r*_*s*_ = −0.13), whereas *Streptococcus gordonii*, linked to FUT2 non-secretors, was associated with increased levels of imidazole propionate (*P* = 6.3 × 10^−40^, *r*_*s*_ = 0.14; Supplementary Table 27). The KEGG functionality module GWAS demonstrated that rs35866622-T, reflecting FUT2 non-secretor status, was reproducibly associated with enhanced Ihk-Irr (virulence regulation) two-component regulatory system (*P* = 6.1 × 10^−11^; Table 2). The relative abundance of *S. gordonii* explained a major part (85%; Supplementary Table 35) of the variance in the activity of Ihk-Irr (virulence regulation) two-component regulatory system, suggesting that this KEGG functionality module is primarily driven by *S. gordonii*.

### Evidence of an effect of BMI on gut microbiota composition

**Fig. 4:**
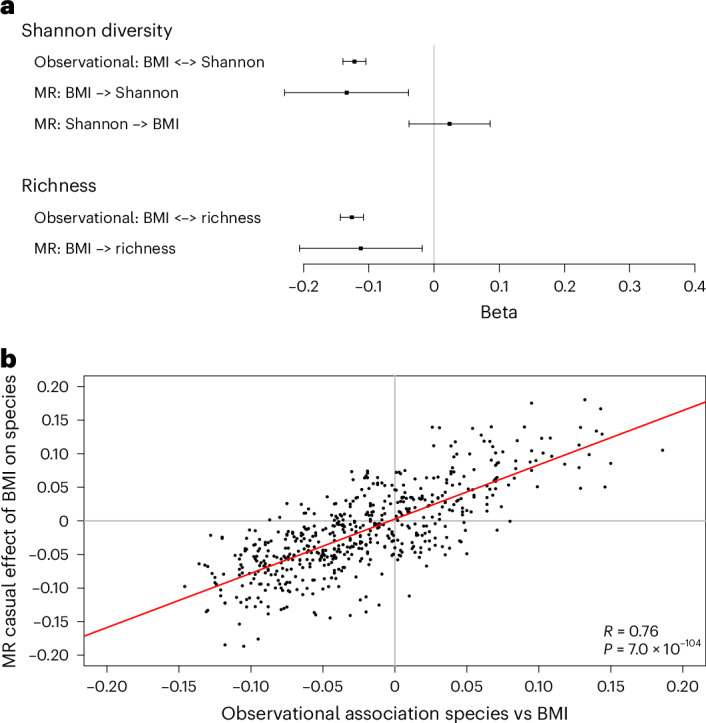
Evidence of a causal effect of BMI on overall gut microbiota composition. **a**, Observational (linear regression) and causal (MR) associations for α-diversity parameters (Shannon index and richness) and BMI. Effect estimates (mean ± 95% confidence intervals) are given as s.d. change in outcome per s.d. increase in exposure. No genetic instrument was available to test the causal effect of richness on BMI. Observational cross-sectional linear regression analyses in HUNT were adjusted for age, sex and library plate (*n* = 12,836). Both the exposures and outcomes were inverse rank transformed before analysis. **b**, Comparison of observational (linear regression analyses) association with causal (MR) association for the effect of BMI on gut microbiota species. The overall observational and causal associations between the 546 evaluated gut microbiota species and BMI were evaluated. Observational associations between gut microbiota species and BMI in HUNT were adjusted for age, sex and library plate and are given as s.d. change in BMI per s.d. change in gut microbiota species (*x*-axis; *n* = 12,840). Effect estimates for the causal effect of BMI on gut microbiota species (*y*-axis) are given as s.d. change in gut microbiota species per s.d. increase in BMI. Both the exposures and outcomes were inverse rank transformed before analyses. The red line represents the correlation of the effect estimates for the observational associations, of all 546 evaluated gut microbiota species, and the effect estimates of the corresponding causal effect of BMI on relative abundance of gut microbiota species. R, Pearson correlation coefficient. *P* value is given for the correlation analysis. The *P* value was calculated using a two-sided *z*-test.

Observational studies have reported associations between gut microbiota composition and BMI, but the underlying causality is unclear^13^. To explore the possible causal associations between BMI and overall gut microbiota composition, we used our current large discovery GWAS data set in HUNT (*n* = 12,652). We first determined the observational association between two α-diversity parameters (Shannon diversity index and species richness) and BMI (Fig. 4a and Supplementary Tables 31 and 32). In models adjusted for age and sex, both Shannon diversity index and richness were inversely associated with BMI. Two-sample MR showed that increased genetically determined BMI reduced both Shannon diversity index and species richness, with similar effect estimates as in the observational linear regression association analyses. Using the single genetic instrument available from the current GWAS for the Shannon diversity index, we did not observe any evidence of reverse causality (Fig. 4a and Supplementary Table 31). However, this analysis is likely underpowered.

Next, we explored the overall observational and causal associations between BMI and the 546 evaluated gut microbiota species. In models adjusted for age and sex, the relative abundances of 129 species (24%) were nominally (*P* < 0.05) directly observationally associated with BMI, and 296 species (54%) were inversely associated with BMI (Supplementary Table 32). Two-sample MR revealed evidence supporting that BMI was nominally causally associated with the relative abundance of 104 species (39 species increased and 65 species decreased with increased BMI, *P* < 0.05; Supplementary Table 32). For these 546 MRs of the effect of BMI on species, the number of nominal significant *P* values observed (*n* = 104) was higher than expected by random (*n* = 27.3; Chi-square, *P* = 3.3 × 10^−49^), suggesting that BMI influences relative abundances of species (Supplementary Table 32). Correlation analyses of the betas for the observational associations of all 546 evaluated species and the betas from the MR of the corresponding causal effect of BMI on the relative abundance of species showed a strong positive correlation (Pearson correlation 0.76, *P* = 7.0 × 10^−104^; Fig. 4b). This finding provides statistical evidence that BMI exerts an overall effect on the relative abundance of the 546 evaluated species, which contributes to the observational associations between gut microbiota species and BMI (Supplementary Note).

## Discussion

The gut microbiota has been associated with human health and disease, but causality is unclear. To unravel host genetics factors influencing gut microbiota composition, we performed a large-scale GWAS including 12,652 participants followed by replication in up to 21,976 participants. We demonstrated a robust contribution of genetic variation to the variability in α-diversity parameters, relative abundance of individual species, and gut microbiota functionality modules in HUNT. We identified 12 reproducible SNP-species associations in six loci, including the known *LCT* and *ABO* loci and the novel *HLA-DQB1*, *MUC12*, *SLC37A2*, and *FUT2* loci. Reproducible genetic signals for KEGG functionality modules were also identified at the *LCT*, *ABO*, and *FUT2* loci. Our follow-up analyses suggested that the identified SNP-species associations might contribute to understanding the underlying pathogenesis of celiac disease and hemorrhoidal disease. In addition, we identified BMI as a determinant of gut microbiota composition.

The substantially higher number of reproducible genetic signals in the present study compared to previous GWASs on gut microbiota composition^2–4,9–11^ is likely due to our large discovery cohort, the use of metagenome sequencing instead of 16S ribosomal RNA gene sequencing, the use of a state-of-the-art microbiome profiling methodology^17^, and using the same standardized high-resolution Genome Taxonomy Database (GTDB) species taxonomy for all included participants (Supplementary Note).

In the present study, the lactase-persistence allele in the *LCT* locus was associated with lower relative abundance of *Bifidobacterium adolescentis*, replicating previous GWAS findings^2–4^. Extending on the previous findings, we showed that the lactase intolerance genetic variant was also associated with increased activity of certain KEGG functionality modules. A likely explanation is enhanced growth of *B. adolescentis* due to the presence of lactase not metabolized by the lactose intolerant hosts. Thereby, there is an increase in the functional pathways that are largely represented by *B. adolescentis* abundance. In addition, these changes in functionality may also be explained by altered dietary habits in combination with altered gut microbiota composition in participants with lactose intolerance.

The SenX3-RegX3 two-component regulatory system was increased in participants with the lactase intolerance genetic variant. This variant is expressed in *Bifidobacterium* and shown to promote expression of the high-affinity phosphate transporter Pst, involved in energy metabolism, which leads to high inorganic phosphate uptake, for example, for ATP synthesis^18^. Gut microbiota species that are more abundant in lactose-intolerant subjects, such as *B. adolescentis*, could have an altered energy metabolism when metabolizing lactose (or other glycans), leading to an increased functionality in the SenX3-RegX3 system (Supplementary Note).

It has been proposed that changes in gut microbiota composition may result in the transition from genetic predisposition to the actual onset of celiac disease characterized by loss of gluten tolerance^19^. However, no gut microbiota species have been reproducibly associated with celiac disease^19^. In the present study, the rs28407950-T allele in the *HLA-DQB1* locus was associated with higher relative abundance of *Agathobacter sp0004342*75 and reduced risk of celiac disease. As celiac disease is a gastrointestinal condition with partly unclear etiology^19,20^, we hypothesized that *Agathobacter sp0004342*75 may play a contributory role. Our cross-sectional observational association analyses in HUNT demonstrated that a high relative abundance of *Agathobacter sp0004342*75 was associated with a low prevalence of celiac disease. There was some evidence from MR that that celiac disease reduces the relative abundance of *Agathobacter sp000434275*, whereas the possible impact of *Agathobacter sp000434275* on celiac disease is unclear (Supplementary Note). In summary, these findings clearly demonstrate that low relative abundance of *Agathobacter sp000434275* is associated with celiac disease, but further studies are warranted to determine causality.

The gut microbiota composition has also been proposed to be involved in the pathogenesis of hemorrhoidal disease, but no gut microbiota species have been reproducibly linked to the disease^21,22^. The present study showed that the rs4556017-T in the *MUC12* locus was associated with a higher relative abundance of *C. cateniformis* and reduced risk of hemorrhoidal disease. We also observed that MUC12, a transmembrane mucin^23^, is highly expressed in colonocytes of the human colon and that the *C. cateniformis* index SNP rs4556017 has an eQTL for *MUC12* in the rectum. A connection between *C. cateniformis* and *MUC12* expression was supported by strong colocalization evidence for a shared causal variant in the *MUC12* locus affecting both the relative abundance of *C. cateniformis* and the expression of *MUC12* in rectum, with increased relative abundance of *C. cateniformis* associated with increased *MUC12* expression in rectum (Supplementary Note).

MUC12 has a transmembrane single-pass domain, a cytoplasmic tail, and an enormous extracellular mucin domain densely decorated with glycans. The glycocalyx of enterocytes and colonocytes is built and composed of transmembrane mucins, such as MUC12 and MUC17, that reach about a micrometer out in the lumen from the cell surface^23^. The small intestinal MUC17-based glycocalyx prevents direct bacterial binding to enterocytes^24^ and may influence the gut microbiota composition^25^. Based on these findings, it is possible that MUC12 in the glycocalyx of colon/rectum might regulate the relative abundance of *C. cateniformis* in feces, which in turn may influence the risk of hemorrhoidal disease. Alternatively, MUC12 might independently affect both the relative abundance of *C. cateniformis* in feces and the risk of hemorrhoidal disease. Further studies are required to delineate the interactions between *C. cateniformis*, MUC12, and hemorrhoidal disease.

The present study observed a novel reproducible genetic signal for *Dysosmobacter sp001916835* in the *SLC37A2* locus. There was some transcriptional support for *SLC37A2*, which encodes a glucose-6-phosphate transporter located in the endoplasmic reticulum^26^, being the gene driving this association. We speculate that host cellular sugar transport/metabolism may influence the host-microbiome interaction. We observed that a higher abundance of *Dysosmobacter sp001916835* was associated with reduced circulating levels of the secondary bile acid isoursodeoxycholate and increased levels of 3-phenylpropionate. Isoursodeoxycholate has been reported to be a marker of poor cardiometabolic health^27^ whereas 3-phenylpropionate indicates high fiber intake^28^, suggesting that the relative abundance of *Dysosmobacter sp001916835* might reflect a healthy diet. However, additional studies are required to characterize the underlying biology connecting *Dysosmobacter sp001916835* with human host genetics.

We observed an interaction between the identified genetic signals at the *ABO* and *FUT2* loci for the relative abundance of *Mediterraneibacter torques*, supporting previous findings^3,4,8^. This interaction is most likely explained by the fact that FUT2 is required for *ABO* antigen expression on the intestinal mucosa. This impacts the relative abundance of certain gut microbiota species dependent on antigens with accessible glycans in non-O blood-type secretors, providing direct energy sources for these species^3^. *M. torques* is a known mucin glycoprotein degrader with strong fucosidase activity^29^, and it is likely that reduced FUT2 activity, resulting in less fucosylated mucin glycans, leads to a lower abundance of *M. torques*^2^.

The index SNP in the *FUT2* locus was also associated with a composite cardiovascular-related parameter. Further separate analyses revealed that an association with high cholesterol and hypertension mainly drove this association. A connection between *Clostridium sp900540255* and high cholesterol was supported by strong evidence for genetic colocalization (Supplementary Note).

Interestingly, FUT2 non-secretors had increased relative abundance of *S. gordonii*, a species normally present in the oral cavity but has also been linked to cardiovascular disease^30^. It was recently demonstrated that the relative abundance of *S. gordonii* in the gut is linked to subclinical coronary atherosclerosis in the well-powered SCAPIS cohort^30^. Gut bacteria have been proposed to affect the development and progression of atherosclerosis via secretion of atherogenic metabolites or through infections local or distal to the atherosclerotic plaque^31^. We observed that high circulating levels of p-cresol sulphate and imidazole propionate, which have been associated with poor cardiometabolic health^31^, were linked via association with certain gut microbiota species to FUT2 non-secretors. Our gut microbiota functionality studies revealed that FUT2 non-secretors were also associated with enhanced Ihk-Irr (virulence regulation) two-component regulatory system. The Ihk/Irr system influences the expression of genes involved in cell wall synthesis and modification, which are critical for the bacteria’s ability to resist destruction by neutrophils^32,33^. We also made the interesting observation that the relative abundance of *S. gordonii* explained a major part of the variance in the activity of the Ihk-Irr (virulence regulation) two-component regulatory system, suggesting that this KEGG functionality module is primarily derived from *S. gordonii*. Therefore, it is likely that FUT2 non-secretors, via increased relative abundance of *S. gordonii*, have enhanced functional capacity of the gut microbiota to evade the host innate immune defense. Further studies are warranted to determine the interaction between FUT2 secretor status, gut microbiota composition and functionality, and circulating metabolites in relation to health outcomes.

Previous observational studies have reported associations between gut microbiota composition and BMI, but the causal direction is unclear^13^. In the present study, two α-diversity parameters (Shannon diversity index and richness) were inversely associated with BMI. MR showed that genetically predicted increased BMI reduced both Shannon diversity index and richness with similar effect sizes as in the cross-sectional linear regression analyses (observational associations). In addition, we demonstrate that BMI exerts an overall effect on the relative abundance of individual gut microbiota species, which contributes to the observational associations between gut microbiota species and BMI. These findings provide evidence of an overall causal effect of BMI, a measure of adiposity, on gut microbiota composition (Supplementary Note).

Strengths of the present study are (i) the large sample size of the discovery cohort, (ii) replication in large independent cohorts, (iii) the use of state-of-the-art metagenome sequencing for gut microbiota analyses, providing high-resolution information on taxonomy and gut microbiota functionality and (iv) the access to large-scale gut microbiota associations with circulating metabolites, which are useful for mechanistic insights. The present study also has limitations. The discovery and replication cohorts mainly included participants of European ancestry living in Nordic countries, and the findings might not be generalizable to populations with other geographical or ancestral origins. The present study provides stronger genetic instruments for a broader panel of species exposures to be used in MR compared with previous GWASs^2–4,9–11^, but these genetic instruments are still relatively weak and often include only a single genome-wide significant independent genetic signal, precluding tests of horizontal pleiotropy. Finally, although several novel loci for gut microbiota species were identified in the present large-scale study, larger meta-analyses of cohorts using the same high-resolution taxonomy are required to further disentangle the genetic architecture by which host genetics regulates the gut microbiota composition and functionality.

In conclusion, our findings support important interactions between host genetics and gut microbiota composition in human health and disease and demonstrate that BMI is a determinant of overall gut microbiota composition.

## Methods

### Discovery cohort: HUNT

The HUNT study is a longitudinal population-based health study conducted in the county of Trøndelag, Norway (Supplementary Note)^34–36^. Among 56,042 participants in the HUNT4 survey (2017-2019), 13,268 participants submitted stool samples for gut microbiome profiling and data from 12,887 of these participants passed the post-metagenome sequencing quality control (Fig. 1a). A total of 12,652 HUNT4 participants of European ancestry had both genetic and gut microbiome data available and were included in the present GWAS (Fig. 1a). The local ethical review board approved the study (regional committee for medical and health research ethics, Midt-Norge; REK-656785), and all participants provided written informed consent.

### Replication cohorts

#### Swedish cohorts

The replication included participants from four population-based Swedish cohorts. Inclusion in the present study was limited to individuals of European ancestry with high-quality metagenomics and genotype data available. The Swedish CArdioPulmonary BioImage Study (SCAPIS)^37^ includes 8,733 participants of European ancestry, aged 50-65, from Malmö and Uppsala with samples collected between 2014 and 2018. The Swedish Infrastructure for Medical Population-Based Life-Course and Environmental Research (SIMPLER; https://www.simpler4health.se/w/sh/en) combines data from the Cohort of Swedish Men and the Swedish Mammography Cohort^38^, including 4,515 men and women from the region of Västmanland (SIMPLER-V) and 981 women from the city of Uppsala (SIMPLER-U) with fecal samples collected between 2011 and 2021. The Malmö Offspring Study (MOS) involves 1,788 adult participants, children, and grandchildren of the Malmö Diet and Cancer (MDC) Study cohort^39^, with samples collected between 2013 and 2017. The association analyses performed in the Swedish cohorts have been approved by the Swedish Ethical Review Authority (DNR 2022-06137-01 and DNR 2024-01992-02). Ethical approval and written informed consent were obtained for the individual Swedish cohorts. Ethical approval for SCAPIS was granted by the Swedish Ethical Review Board (DNR 2010-228-31 M) and all participants gave written informed consent. The SIMPLER studies received approval from the Swedish Ethical Review Board (DNR 2009/2066-32, DNR 2009/1935-32, DNR 2010/0148-32, DNR 2014/892-31/3), and all participants gave written informed consent. The MOS study received approval from the Ethics Review Committee of Lund University (DNR 2012-594), and all participants gave written informed consent.

#### FINRISK

For four identified SNP-species associations (but no SNP-KEGG functionality module), data for replication was also publicly available from the FINRISK cohort (*n* = 5,959)^4^. The participants in the FINRISK cohort were analyzed using shallow metagenome sequencing as previously described^4^ and the Genome Taxonomy Database (GTDB) was used for annotation of taxa included in the published FINRISK GWAS^4^.

### Gut microbiome profiling - HUNT

#### Metagenome sequencing of HUNT samples

Stool collection and DNA isolation and quantification have been performed using a standardized procedure, as previously described^13^ before sequencing and microbiome profiling at Clinical Microbiomics in Denmark (Fig. 1a and Supplementary Note)^17^.

The enzymatic fragmentation of DNA and library construction was conducted on a Tecan DreamPrep NGS automation system using the Celero EZ DNA-seq Core Module. A DNA sample volume of 10 μl was used. To ensure that the maximum amount of 500 ng input DNA recommended by the manufacturer was not exceeded, all samples with DNA concentrations >50 ng μl^−1^ were diluted to 30 ng μl^−1^. The fragmented DNA was amplified using PCR. Short and long DNA fragments were removed using double-sided magnetic bead size selection (AMPure XP, Beckman Coulter, reference A63882). Adapter sequences from Celero 96-Plex Adaptor Plate were added to each sample during library construction. The final concentration for each library was quantified by Tecan Infinite F Nano+ Plate Reader using NuQuant NGS Library Quantification Module. Qubit and TapeStation were used to determine the concentration of the final library before sequencing at 2 × 150 bp on an Illumina NovaSeq 6000. Samples were sequenced to an average depth of 22.9 million read pairs per sample. For each sample, more than 85% (mean ≥ 30 = 93.8%) of the bases had a Phred quality score of ≥30.

#### Gene catalog and species definitions

Gut microbiome profiling was performed using the Clinical Microbiomics Human Microbiome Profiler (CHAMP) pipeline, which uses the GTDB r214 for taxonomic annotation of prokaryotes^17^. Clinical Microbiomics gave species missing from the GTDB r214 database a unique species ID (“hMGS”). The HMR05 catalog used in the present study was based on 30,382 samples from nine human body sites, including prokaryotic metagenome-assembled genomes (MAGs) mainly from the Unified Human Gastrointestinal Genome collection^40^ and the Early-Life Gut Genomes catalog^41^. In addition, genome assemblies from NCBI and PATRIC were added to capture otherwise missing species of interest (human-associated pathogens, probiotics, food ingredients and species relevant for benchmarking). MAGs were clustered by species using the Genome Taxonomy Database Toolkit (GTDB-Tk release R214), whereas unannotated MAGs were clustered at 95% identity using FastANI. The catalog included 6,809 microorganisms.

Human-relevant eukaryotic species were manually identified from various sources, including an analysis of gut fungal species^42^, publicly available lists of pathogens, the eukaryotes profiled by MetaPhlAn 4 (ref. ^43^), and various species relevant for benchmarking. The result was 2,740 genomes representing 244 species.

For MAGs not obtained from publicly available MAG collections, reads were host-filtered, trimmed, and assembled into contigs with Megahit (v.1.2.9)^44^ or metaSPAdes (v.3.15.5)^45^ and then binned using VAMB (v.3.0.6)^46^. MAGs were considered high-quality if they had >90% completeness and < 5% contamination based on CheckM2 (v.2022-07-19) and passed the GUNC chimerism test (v.1.0.5)^47^. All MAGs were taxonomically annotated using GTDB-Tk (v.2.3.0)^48^ with GTDB database (v.r214)^49^. To combine MAGs from multiple VAMB batches and MAG collections, MAGs annotated to the same species were merged into species clusters. MAGs without GTDB-Tk species-level annotations were merged with each other or with existing species clusters at 95% identity (dRep^50^; FastANI^51^). This resulted in 6,567 prokaryotic species clusters, 10% of which were unannotated at the species level. We used a three-step clustering approach to derive a pan-genome catalog for each species. First, genes were clustered with MMseqs2 (v.14)^52^ with 98% identity and 90% bi-directional coverage. Second, the representatives from the first iteration were clustered with MMseqs2 with 95% identity and 90% bi-directional coverage. Representatives of the second iteration were chosen as the ones with highest cardinality from the first iteration. Third, the second iteration representatives were clustered with cd-hit (cd-hit-est, v.4.8.1)^53^ with 95% identity and 90% coverage of the shorter sequence. Genes shorter than 100 bp or with species prevalence < 1% were discarded. For prokaryotes and eukaryotes separately, the entire set of pangenomes was then clustered with MMseqs2 with 97% identity and 90% bi-directional coverage to obtain between-species clusters. The pan-genomes from prokaryotic (*n* = 6,567) and eukaryotic (*n* = 244) species were merged into a final catalog of 25,761,278 genes.

To enable quantification of each species in the database, up to 250 signature genes were selected for each species based on core genes (≥60% prevalence in species MAGs) with a length ≥200 bp and ≤20 kb. Furthermore, signature genes were required to be species unique, with no alignments of 100 bp with >97% sequence-identity to other genes in the catalog. However, if fewer than 20 genes meeting this criterion were available for a species, then genes with segments >200 bp without alignments to other genes were used, and non-unique segments of these genes were masked.

#### HUNT sequencing data preprocessing

Read pairs mapped to the human reference genome GRCh38.p14 were removed using Bowtie2 (v2.4.2)^54^. Reads were then trimmed to remove adapters and bases with a Phred score below 30 using AdapterRemoval (v. 2.3.1)^55^. Host-filtered read pairs with both lengths ≥100 bp, defined as high-quality nonhost (HQNH) reads, were retained.

#### Mapping HUNT sample reads to the gene catalog

HQNH reads from the HUNT samples were mapped to the gene catalog using BWA mem (v. 0.7.17)^56^. An individual read was considered uniquely mapped to a gene if the mapping quality (MAPQ) was ≥20 and the read aligned with ≥95% identity over ≥100 bp. However, if >10 bases of the read did not align with the gene or extend beyond the gene, the read was considered unmapped. Reads meeting the alignment length and identity criteria but not the MAPQ threshold were considered multi-mapped. Each read pair was counted as either (1) uniquely mapped to a specific gene, if one or both individual reads were uniquely mapped to a gene, or (2) multi-mapped, if neither read was uniquely mapped, and at least one was multi-mapped, or (3) unmapped, if both individual reads were unmapped. If the two reads were each uniquely mapped to a different gene, the gene mapped by read one was counted but not the gene mapped by read two. A gene count table was created with the number of uniquely mapped read pairs for each gene.

#### Species relative abundance calculation

The relative abundance of each species (MAGs) was calculated based on the species signature genes with observed read counts within the expected 99% quantile and normalized sample-wise so that the total abundance of all species was summed to 100%. The expected read counts for signature genes in each species in each sample were modelled with a negative binomial distribution as follows. First, if ≥50 of the signature genes for a species had non-zero read counts and ≥99% of genes were expected to have non-zero read counts given the total read count for that species, then signature genes with zero reads were ignored in that sample. Second, the expected 99% quantile (between 0.5% and 99.5%) of read counts was calculated for each gene based on a negative binomial distribution with a mean proportional to the effective gene length (accounting for read length and mapping alignment criteria) and dispersion defined as log_2_ (effective gene length). The abundance of each species was then calculated as the mean read count normalized by effective gene length based on reads mapping to signature genes with observed read counts within the expected 99% quantile. Species abundances were set to zero if less than five genes with non-zero read counts were within the 99% quantile. Furthermore, species with < 66% of genes with non-zero read count within the 99% quantile were set to zero, unless the median abundance of signature genes was non-zero, in which case the median gene-length-corrected abundance of non-zero genes was used. Abundances were then normalized sample-wise such that all species’ total abundance was 100% (Supplementary Note).

In addition, for the estimation of α-diversity measures, rarefied species abundance profiles were calculated by random sampling, without replacement, of a fixed number of signature gene counts per sample and following the procedure described above. In HUNT, 164,245 signature gene counts were sampled for the rarefied dataset. After profiling the rarefied data set, the α-diversity measures (Shannon diversity index and richness) were calculated using rarefied species relative abundances with the diversity function of the vegan R package.

In the HUNT gut microbiota cohort, on average, 84% of the high-quality microbiome reads from a sample were mapped to the Clinical Microbiomics HMR05 gene catalog, and on average, 459 gut microbiota species were detected per sample. A total of 12,887 high-quality samples passed the post-metagenome sequencing quality control in HUNT, with 4,870 gut microbiota species present in at least one sample (Fig. 1a). For the subsequent association studies, the relative abundance of species and α-diversity measures were inverse rank normal transformed.

#### Functional annotation and profiling

EggNOG-mapper (v. 2.1.7, Diamond mode)^57^ was used to map prokaryotic genes in the gene catalog to the EggNOG orthologous groups database (v. 5.0)^58^ and Kyoto Encyclopedia of Genes and Genomes (KEGG) Orthology (KO) database. Eukaryotic genes were annotated using KofamScan^59^. Functional potential profiles based on KOs were calculated as the proportion of the total gene abundance mapped to a given KO.

KEGG modules (v. 78.2)^60^ were defined as a set of KOs that enable a specific function or pathway. Functional potential profiles based on KEGG modules were generated from the species profiles in HUNT. For this, we identified the set of species associated with each of the KEGG modules by following three criteria: (1) a species was associated with a KEGG module if it included at least 2/3 of the genes encoding the proteins/enzymes needed to complete the functionality of the module; (2) if a module had alternative reaction paths, only one of these was required to be 2/3 complete; and (3) for modules with three or fewer steps, all steps were required to be comprised in the given species. KEGG module profiles based on relative abundances were then calculated by adding the relative abundances of each species fulfilling the criteria for being associated with a given KEGG module.

### Gut microbiome profiling: Swedish cohorts

#### SCAPIS and MOS

DNA extraction, quality control, and library preparation of metagenomic DNA for SCAPIS and MOS have been performed by Clinical Microbiomics A/S and described in detail before^30^. Libraries from stool DNA were sequenced using the Illumina NovaSeq 6000 instrument using 2 × 150 bp paired-end reads, generating on average 26.0 and 25.3 million read pairs, respectively in SCAPIS and MOS with 97.8% of the sequenced bases having Phred quality score >20.

#### SIMPLER (SIMPLER-V and SIMPLER-U)

Stool samples were thawed, and a small portion was aliquoted and combined with 800 μl DNA/RNA Shield. These aliquots were sent to the Centre for Translational Microbiome Research at the Karolinska Institutet in Stockholm for metagenomic DNA extraction and sequencing, conducted during 2022 and 2023. The DNA from the samples was extracted using the MagPure Stool kit. The genomic DNA was then fragmented and used to construct libraries using the MGIEasy FS DNA Library Prep Set kit. The prepared DNA libraries were evaluated using a TapeStation D1000 kit, and their quantity was determined by a QuantIT High Sensitivity dsDNA Assay on a Tecan Spark. The pooled libraries were circularized using the MGI Easy Circularization kit and sequenced with 2 × 150 bp paired-end reads on the DNBSEQ G400 or T7 sequencing instrument, following the manufacturer’s instructions, resulting in an average yield of 51 million reads per sample.

Microbial taxonomy profiling was performed for all four Swedish replication cohorts at Clinical Microbiomics using the CHAMP profiler based on the Human Microbiome Reference HMR05 catalog in a similar manner to that described for HUNT above.

### Genotyping and imputation: HUNT

HUNT participants were genotyped using Illumina HumanCoreExome arrays and genotype data were imputed to the Human Reference Consortium (HRC) 1.1 panel (Supplementary Note)^61^.

### Genotyping and imputation: Swedish cohorts

The Swedish cohorts were genotyped using Illumina GSA arrays and genotype data were imputed to the HRC 1.1 panel.

### GWAS of gut microbiota species

We performed GWAS of the relative abundance of 546 gut microbiota species (prevalence >30%; primary outcomes) in 12,652 HUNT4 participants of European ancestry using linear ridge regression under an additive genetic model for each variant as implemented by REGENIE (v.3.4.1)^62^. Variants with a MAF < 1% or INFO < 0.3 were excluded from the analyses, leaving 7,971,623 common and low-frequency genetic variants for testing. Before analysis, we applied an inverse rank normal transformation of the relative abundance of each gut microbiota species. Age, sex, genotyping batch, library plate, and the first ten principal components of ancestry were included as covariates in the analyses. To be taken forward to replication efforts, we required a *P* < 1.3 × 10^−10^ (genome-wide significance adjusted for number of effective tests, which we estimated to be 391)^3^. We selected the index SNP for each species in each locus. Among the 13 selected SNP-species associations, 12 passed a conservative threshold of *P* < 9.2 × 10^−11^, adjusting for all 546 gut microbiota species analyses, whereas the last one selected passed the significance level adjusting for number of effective tests (*P* < 1.3 × 10^−10^) in HUNT (Table 1).

To test for multiple independent association signals within each locus, we performed stepwise conditional regression analyses in each identified locus. We included the same covariates and inverse-rank transformation of the variables as in the main analysis, but we added the index variant in the previous step as a covariate for each consecutive step.

### Replication of identified SNP–species signals

We took 13 SNP–species associations discovered in HUNT forward for replication in five Nordic replication cohorts. We tested all SNP-species associations in the four meta-analyzed Swedish replication cohorts (SCAPIS, *n* = 8,733; SIMPLER-V, *n* = 4,515; SIMPLER-U, *n* = 981; MOS, *n* = 1,788; total sample size, *n* = 16,017). For four identified SNP-species associations, data for replication was publicly available from the Finnish FINRISK cohort (*n* = 5,959)^4^. For successful replication, concordant direction of effect in the combined replication data set (*n* = 16,017-21,976) and *P* < 3.8 × 10^−3^ (Bonferroni correction for 13 comparisons) were required.

Meta-analyses were performed either with fixed-effect inverse-variance weighted or sample size weighted meta-analysis using METAL (v. 2011-03-25)^63^. For meta-analyses including the FINRISK study, sample size weighted meta-analysis was used because the effect sizes were on a different scale than the other cohorts (HUNT and the Swedish cohorts used standardized inverse rank transformed relative abundance of species, whereas FINRISK used standardized center log-transformed relative abundance of species).

### Sensitivity analyses considering antibiotic treatment

In sensitivity analyses, we excluded participants with recent antibiotic use defined as a dispensed prescription (Anatomical Therapeutic Chemical codes J01 and J04; *n* = 1,046) up to 3 months before the delivery of the fecal sample kit (Supplementary Note).

### GWAS for KEGG functionality modules and α-diversity measures

To further follow up on the results from the primary GWAS on gut microbiota species, we used the above described GWAS approach and performed GWAS of 461 gut microbiota KEGG functionality modules (prevalence ≥30%; Supplementary Table 24) and two α -diversity measures (Shannon diversity index and richness (number of observed species) calculated from rarefied data with a rarefication target of 164,245 signature gene counts). Shannon diversity index was calculated using the R package vegan (v.2.6-4).

For KEGG functionality modules, we required a *P* < 4.9 × 10^−10^ (genome-wide significant threshold adjusted for the number of effective tests, which was estimated to be 102)^3^ to be selected for replication in the Swedish cohorts (Supplementary Fig. 4a). We selected the most significant genetic signal for each KEGG functionality module in each locus (Table 2). Tests for replication were performed in the Swedish cohorts for eight SNP-KEGG functionality module associations identified in the HUNT discovery cohort (Supplementary Fig. 4a). For successful replication, concordant direction of effect in the replication data and *P* < 6.25 × 10^−3^ (Bonferroni correction for eight comparisons) were required (Supplementary Fig. 4a).

### Definition of celiac disease cases in HUNT

Participants with celiac disease were identified through serological screening and linkage to hospital journal records and the Norwegian Patient Registry^64^. The 240 celiac disease participants with available fecal samples from HUNT4 were included in the present study. The associations between the relative abundance of a gut microbiota species and celiac disease prevalence were determined by logistic regression, adjusting for age, sex and library plate.

### SNP heritability

#### SNP heritability using GCTA

We estimated the narrow-sense (additive) SNP heritability (V_g_/V_p_ ± SE, where V_g_ is the variance explained by the SNPs and V_p_ is the total phenotypic variance) of the α-diversity parameters (Shannon diversity index and richness), and of the relative abundances of gut microbiota species and KEGG modules in HUNT, using genome-wide complex trait analysis (GCTA) (v. 1.94.1)^65,66^. We first created a genetic relationship matrix (GRM) based on 365,943 genotyped autosomal variants in 8,593 unrelated (no first- or second-degree relatives as estimated by KING v.2.3.2)^67^ HUNT4 participants. Secondly, we used the GRM with GCTA-GREML (genomic relatedness-based restricted maximum-likelihood) to estimate the phenotypic variance explained by the genetic variants for relative abundance of each investigated parameter after inverse rank transformation. For each estimate, we included age, sex, genotyping batch and library plate as covariates in the analysis.

#### Heritability estimates using LD score regression

As an alternative method to estimate the heritability of α-diversity parameters (Shannon index and richness) and the relative abundance of gut microbiota species, we used LD score regression as implemented in the LD score tool available on Github (https://github.com/bulik/ldsc)^68^. The LD score regression analyses were restricted to HapMap3 SNPs with MAF > 5% in the 1000 Genomes European reference population. We used precalculated LD scores from the same reference panel (https://data.broadinstitute.org/alkesgroup/LDSCORE/).

### MR

As exposures in the two-sample MR, we used genetic instruments for the relative abundance of gut microbiota species and Shannon diversity index, derived from the current discovery GWAS, selected human diseases, identified in our PheWAS, derived from publicly available GWAS data sets (celiac disease^69^, hemorrhoidal disease^70^, cardiovascular-related outcomes^71^), and BMI^72^. We only selected variants with a MAF > 1% and *P* < 5 × 10^−8^. We selected instruments with *r*^2^ < 0.01 (based on the European populations in LDlink)^73^ to ensure little correlation between instruments. The variance explained (*R*^2^) and F statistic for the genetic instruments were estimated from the respective GWAS summary statistics (Supplementary Table 25). For exposures with multiple genetic instruments, we applied the inverse variance weighted method using fixed or random effects depending on the Cochran’s Q statistic test of heterogeneity. We then used the MR-Egger regression as a sensitivity analysis to test for possible directional horizontal pleiotropy^74^. In further sensitivity analyses, we used the weighted median MR method. For exposures with only one genetic instrument, we estimated the Wald ratio. The MR analyses were conducted using the R package MendelianRandomization^75^.

### Colocalization

To assess if any of the identified gut microbiota species loci were consistent with having shared causal variants with selected human diseases or tissue-specific eQTLs of interest, we combined their GWAS summary statistics and performed a Bayesian colocalization analysis as implemented in the R package coloc (Supplementary Note)^76^.

### Associations between gut microbiota species and circulating metabolites in the SCAPIS cohort

For mechanistic insights, we also evaluated the associations for the gut microbiota species with replicated genetic signals with circulating metabolites, analyzed using the Metabolon platform in the Swedish SCAPIS cohort^77^. We considered the top three annotated circulating metabolites associated with each species with replicated SNP-species GWAS findings (Supplementary Table 27). To investigate the association of genetic variants and species abundance with plasma metabolite levels, we conducted partial Spearman’s rank correlations adjusted for age, sex, place of birth and metabolomics delivery batch.

### Dual RNAscope and immunohistochemistry of MUC12 in the human sigmoid colon

Biopsies from the sigmoid colon were collected from patients (>18 years) with normal intestinal macroscopy who were referred for colonoscopy to the Sahlgrenska University Hospital, Gothenburg. The protocol complied with the Declaration of Helsinki and was approved by the Research Ethical Committee in Gothenburg (ethical permission 2020-03196). All patients gave written informed consent. Collected biopsies were fixed in 4% paraformaldehyde and embedded in paraffin.

An anti-MUC12-S2 polyclonal rabbit antibody was raised against the peptide DYTLEYEELFENLAEIVKAKIMNEC. Fluorescent in situ hybridization (FISH) on the tissue described above was performed using the Multiplex Fluorescent Detection Reagent v2 (ACD; 323110), following the manufacturer’s standard RNAscope protocols. The probe Hs-MUC12-O1-C1 (1569341-C1) was used to detect *MUC12*, with fluorescent signals visualized using the TSA Plus Cyanine 5 system (PerkinElmer, NEL705A001KT). Immediately after the *MUC12* RNAscope FISH detection, immunofluorescence was performed on the same tissue section. Blocking serum was applied and incubated for 1 h before the addition of primary antibodies against either EpCAM (1:250, Abcam, ab71916, lot #1076051-3) and MUC2-C3 (1:100, GeneTex, GTX100664, lot #44818) or EpCAM and MUC12-S2 (1:250), which were diluted in blocking serum and left to incubate overnight at 4 °C. Subsequently, secondary antibodies, either Goat anti-Mouse IgG, IgM (H + L) Secondary Antibody, Alexa Fluor 488 (1:400, ThermoFisher, catalog #A10680, lot #1664758) or Cy3 AffiniPure Donkey Anti-Rabbit IgG (H + L) (1:400, Jackson ImmunoResearch, catalog #711-165-152, lot #171768) were applied and incubated for 1 h at room temperature, after which the tissue was counterstained with DAPI and imaged using a Nikon Spinning Disk system.

### Reporting summary

Further information on research design is available in the Nature Portfolio Reporting Summary linked to this article.

## Online content

Any methods, additional references, Nature Portfolio reporting summaries, source data, extended data, supplementary information, acknowledgements, peer review information; details of author contributions and competing interests; and statements of data and code availability are available at 10.1038/s41588-026-02502-4.

## Supplementary information

Supplementary Information Supplementary Note and Supplementary Figs. 1–8.

Reporting Summary

Supplementary Table 1–35 Supplementary Tables 1–35.

## Data Availability

Individual-level data from HUNT can be accessed by, or in collaboration with, a Norwegian principal investigator. Researchers can apply for HUNT data access from HUNT Research Centre (https://www.ntnu.edu/hunt) if they have obtained project approval from the Regional Committee for Medical and Health Research Ethics (REC). Information on the application and conditions for data access is available at https://www.ntnu.edu/hunt/data. For the replication cohorts, the genetic data used in the SCAPIS, SIMPLER and MOS board are not shared publicly due to confidentiality. Data will be shared upon reasonable request after permission from the Swedish Ethical Review Authority (https://etikprovningsmyndigheten.se) and from the respective cohort boards (https://www.scapis.org/data-access, https://www.simpler4health.se and https://www.malmo-kohorter.lu.se/malmo-offspring-study-mos). Summary statistics of the discovery GWAS are available at the GWAS Catalog under study accession numbers GCST90666541–GCST90667549 (https://www.ebi.ac.uk/gwas). Genome Taxonomy Database Toolkit (GTDB-Tk release R214) can be found at https://gtdb.ecogenomic.org.

## Code availability

All analyses have been performed using publicly available software, tools, packages and databases as given in Methods. Most analyses have been performed using R v4.1.1 or higher (https://cran.r-project.org/).

## References

[1] Wilmes, P. et al. The gut microbiome molecular complex in human health and disease. *Cell Host Microbe* **30**, 1201–1206 (2022). 36108612 10.1016/j.chom.2022.08.016

[2] Kurilshikov, A. et al. Large-scale association analyses identify host factors influencing human gut microbiome composition. *Nat. Genet.* **53**, 156–165 (2021). 33462485 10.1038/s41588-020-00763-1 PMC8515199

[3] Lopera-Maya, E. A. et al. Effect of host genetics on the gut microbiome in 7,738 participants of the Dutch Microbiome Project. *Nat. Genet.* **54**, 143–151 (2022). 35115690 10.1038/s41588-021-00992-y

[4] Qin, Y. et al. Combined effects of host genetics and diet on human gut microbiota and incident disease in a single population cohort. *Nat. Genet.* **54**, 134–142 (2022). 35115689 10.1038/s41588-021-00991-z PMC9883041

[5] Gomaa, E. Z. Human gut microbiota/microbiome in health and diseases: a review. *Antonie Van Leeuwenhoek* **113**, 2019–2040 (2020). 33136284 10.1007/s10482-020-01474-7

[6] Goodrich, J. K. et al. Genetic determinants of the gut microbiome in UK twins. *Cell Host Microbe* **19**, 731–743 (2016). 27173935 10.1016/j.chom.2016.04.017 PMC4915943

[7] Goodrich, J. K. et al. Human genetics shape the gut microbiome. *Cell* **159**, 789–799 (2014). 25417156 10.1016/j.cell.2014.09.053 PMC4255478

[8] Ruhlemann, M. C. et al. Genome-wide association study in 8,956 German individuals identifies influence of ABO histo-blood groups on gut microbiome. *Nat. Genet.* **53**, 147–155 (2021). 33462482 10.1038/s41588-020-00747-1

[9] Bonder, M. J. et al. The effect of host genetics on the gut microbiome. *Nat. Genet.* **48**, 1407–1412 (2016). 27694959 10.1038/ng.3663

[10] Wang, J. et al. Genome-wide association analysis identifies variation in vitamin D receptor and other host factors influencing the gut microbiota. *Nat. Genet.* **48**, 1396–1406 (2016). 27723756 10.1038/ng.3695 PMC5626933

[11] Turpin, W. et al. Association of host genome with intestinal microbial composition in a large healthy cohort. *Nat. Genet.* **48**, 1413–1417 (2016). 27694960 10.1038/ng.3693

[12] Vujkovic-Cvijin, I. et al. Host variables confound gut microbiota studies of human disease. *Nature* **587**, 448–454 (2020). 33149306 10.1038/s41586-020-2881-9 PMC7677204

[13] Grahnemo, L. et al. Cross-sectional associations between the gut microbe *Ruminococcus gnavus* and features of the metabolic syndrome. *Lancet Diabetes Endocrinol.* **10**, 481–483 (2022). 35662399 10.1016/S2213-8587(22)00113-9

[14] Ochoa, D. et al. The next-generation Open Targets Platform: reimagined, redesigned, rebuilt. *Nucleic Acids Res.* **51**, D1353–D1359 (2023). 36399499 10.1093/nar/gkac1046 PMC9825572

[15] Noguchi, S. et al. FANTOM5 CAGE profiles of human and mouse samples. *Sci. Data* **4**, 170112 (2017). 28850106 10.1038/sdata.2017.112 PMC5574368

[16] Kashyap, P. C. et al. Genetically dictated change in host mucus carbohydrate landscape exerts a diet-dependent effect on the gut microbiota. *Proc. Natl Acad. Sci. USA* **110**, 17059–17064 (2013). 24062455 10.1073/pnas.1306070110 PMC3800993

[17] Pita, S. et al. CHAMP delivers accurate taxonomic profiles of the prokaryotes, eukaryotes, and bacteriophages in the human microbiome. *Front. Microbiol.* **15**, 1425489 (2024). 39483755 10.3389/fmicb.2024.1425489 PMC11524946

[18] An, H. et al. Integrated transcriptomic and proteomic analysis of the bile stress response in a centenarian-originated probiotic *Bifidobacterium longum* BBMN68. *Mol. Cell. Proteomics* **13**, 2558–2572 (2014). 24965555 10.1074/mcp.M114.039156 PMC4188986

[19] Matera, M. & Guandalini, S. How the microbiota may affect celiac disease and what we can do. *Nutrients* **16**, 1882 (2024). 38931237 10.3390/nu16121882 PMC11206804

[20] Caio, G. et al. Celiac disease: a comprehensive current review. *BMC Med.* **17**, 142 (2019). 31331324 10.1186/s12916-019-1380-z PMC6647104

[21] Palumbo, V. D. et al. Altered gut microbic flora and haemorrhoids: could they have a possible relationship? *J. Clin. Med.* **12**, 2198 (2023). 36983199 10.3390/jcm12062198 PMC10054427

[22] Yang, F., Lan, Z., Chen, H. & He, R. Causal associations between human gut microbiota and hemorrhoidal disease: A two-sample Mendelian randomization study. *Medicine (Baltimore)* **103**, e37599 (2024). 38552035 10.1097/MD.0000000000037599 PMC10977532

[23] Pelaseyed, T. et al. The mucus and mucins of the goblet cells and enterocytes provide the first defense line of the gastrointestinal tract and interact with the immune system. *Immunol. Rev.* **260**, 8–20 (2014). 24942678 10.1111/imr.12182 PMC4281373

[24] Layunta, E., Javerfelt, S., Dolan, B., Arike, L. & Pelaseyed, T. IL-22 promotes the formation of a MUC17 glycocalyx barrier in the postnatal small intestine during weaning. *Cell Rep.* **34**, 108757 (2021). 33596425 10.1016/j.celrep.2021.108757

[25] Layunta, E. et al. MUC17 is an essential small intestinal glycocalyx component that is disrupted in Crohn’s disease. *JCI Insight* **10**, e181481 (2024). 39699961 10.1172/jci.insight.181481 PMC11948581

[26] Ng, P. Y. et al. Sugar transporter Slc37a2 regulates bone metabolism in mice via a tubular lysosomal network in osteoclasts. *Nat. Commun.* **14**, 906 (2023). 36810735 10.1038/s41467-023-36484-2 PMC9945426

[27] Louca, P. et al. The secondary bile acid isoursodeoxycholate correlates with post-prandial lipemia, inflammation, and appetite and changes post-bariatric surgery. *Cell. Rep. Med.* **4**, 100993 (2023). 37023745 10.1016/j.xcrm.2023.100993 PMC10140478

[28] Wang, Z. et al. Gut microbiota and blood metabolites related to fiber intake and type 2 diabetes. *Circ. Res.* **134**, 842–854 (2024). 38547246 10.1161/CIRCRESAHA.123.323634 PMC10987058

[29] Schaus, S. R. et al. *Ruminococcus torques* is a keystone degrader of intestinal mucin glycoprotein, releasing oligosaccharides used by *Bacteroides thetaiotaomicron*. *mBio* **15**, e0003924 (2024). 38975756 10.1128/mbio.00039-24 PMC11323728

[30] Sayols-Baixeras, S. et al. Streptococcus species abundance in the gut is linked to subclinical coronary atherosclerosis in 8973 participants from the SCAPIS cohort. *Circulation* **148**, 459–472 (2023). 37435755 10.1161/CIRCULATIONAHA.123.063914 PMC10399955

[31] Jonsson, A. L. & Backhed, F. Role of gut microbiota in atherosclerosis. *Nat. Rev. Cardiol.* **14**, 79–87 (2017). 27905479 10.1038/nrcardio.2016.183

[32] Voyich, J. M. et al. Engagement of the pathogen survival response used by group A *Streptococcus* to avert destruction by innate host defense. *J. Immunol.* **173**, 1194–1201 (2004). 15240710 10.4049/jimmunol.173.2.1194

[33] Voyich, J. M., Musser, J. M. & DeLeo, F. R. *Streptococcus pyogenes* and human neutrophils: a paradigm for evasion of innate host defense by bacterial pathogens. *Microbes Infect.* **6**, 1117–1123 (2004). 15380782 10.1016/j.micinf.2004.05.022

[34] Krokstad, S. et al. Cohort profile: the HUNT study, Norway. *Int. J. Epidemiol.* **42**, 968–977 (2013). 22879362 10.1093/ije/dys095

[35] Asvold, B. O. et al. Cohort profile update: the HUNT study, Norway. *Int. J. Epidemiol.* **52**, e80–e91 (2023). 35578897 10.1093/ije/dyac095 PMC9908054

[36] Naess, M. et al. Data resource profile: the HUNT Biobank. *Int. J. Epidemiol.* **53**, dyae073 (2024). 38836303 10.1093/ije/dyae073 PMC11150882

[37] Bergstrom, G. et al. The Swedish CArdioPulmonary BioImage Study: objectives and design. *J. Intern. Med.* **278**, 645–659 (2015). 26096600 10.1111/joim.12384 PMC4744991

[38] Warensjo Lemming, E. et al. Dietary fatty acids and incident hip fractures in cohorts of women and men. A relative validation and follow-up study. *J. Nutr. Health Aging.* **28**, 100247 (2024). 38669838 10.1016/j.jnha.2024.100247 PMC12433805

[39] Brunkwall, L. et al. The Malmo Offspring Study (MOS): design, methods and first results. *Eur. J. Epidemiol.* **36**, 103–116 (2021). 33222051 10.1007/s10654-020-00695-4 PMC7847466

[40] Almeida, A. et al. A unified catalog of 204,938 reference genomes from the human gut microbiome. *Nat. Biotechnol.* **39**, 105–114 (2021). 32690973 10.1038/s41587-020-0603-3 PMC7801254

[41] Zeng, S. et al. A compendium of 32,277 metagenome-assembled genomes and over 80 million genes from the early-life human gut microbiome. *Nat. Commun.* **13**, 5139 (2022). 36050292 10.1038/s41467-022-32805-z PMC9437082

[42] Nash, A. K. et al. The gut mycobiome of the Human Microbiome Project healthy cohort. *Microbiome* **5**, 153 (2017). 29178920 10.1186/s40168-017-0373-4 PMC5702186

[43] Blanco-Miguez, A. et al. Extending and improving metagenomic taxonomic profiling with uncharacterized species using MetaPhlAn 4. *Nat. Biotechnol.* **41**, 1633–1644 (2023). 36823356 10.1038/s41587-023-01688-w PMC10635831

[44] Li, D., Liu, C. M., Luo, R., Sadakane, K. & Lam, T. W. MEGAHIT: an ultra-fast single-node solution for large and complex metagenomics assembly via succinct de Bruijn graph. *Bioinformatics* **31**, 1674–1676 (2015). 25609793 10.1093/bioinformatics/btv033

[45] Nurk, S., Meleshko, D., Korobeynikov, A. & Pevzner, P. A. metaSPAdes: a new versatile metagenomic assembler. *Genome Res.* **27**, 824–834 (2017). 28298430 10.1101/gr.213959.116 PMC5411777

[46] Nissen, J. N. et al. Improved metagenome binning and assembly using deep variational autoencoders. *Nat. Biotechnol.* **39**, 555–560 (2021). 33398153 10.1038/s41587-020-00777-4

[47] Orakov, A. et al. GUNC: detection of chimerism and contamination in prokaryotic genomes. *Genome Biol.* **22**, 178 (2021). 34120611 10.1186/s13059-021-02393-0 PMC8201837

[48] Chaumeil, P. A., Mussig, A. J., Hugenholtz, P. & Parks, D. H. GTDB-Tk v2: memory friendly classification with the genome taxonomy database. *Bioinformatics* **38**, 5315–5316 (2022). 36218463 10.1093/bioinformatics/btac672 PMC9710552

[49] Parks, D. H. et al. GTDB: an ongoing census of bacterial and archaeal diversity through a phylogenetically consistent, rank normalized and complete genome-based taxonomy. *Nucleic Acids Res.* **50**, D785–D794 (2022). 34520557 10.1093/nar/gkab776 PMC8728215

[50] Olm, M. R., Brown, C. T., Brooks, B. & Banfield, J. F. dRep: a tool for fast and accurate genomic comparisons that enables improved genome recovery from metagenomes through de-replication. *ISME J.* **11**, 2864–2868 (2017). 28742071 10.1038/ismej.2017.126 PMC5702732

[51] Jain, C., Rodriguez, R. L., Phillippy, A. M., Konstantinidis, K. T. & Aluru, S. High throughput ANI analysis of 90 K prokaryotic genomes reveals clear species boundaries. *Nat. Commun.* **9**, 5114 (2018). 30504855 10.1038/s41467-018-07641-9 PMC6269478

[52] Steinegger, M. & Soding, J. Clustering huge protein sequence sets in linear time. *Nat. Commun.* **9**, 2542 (2018). 29959318 10.1038/s41467-018-04964-5 PMC6026198

[53] Fu, L., Niu, B., Zhu, Z., Wu, S. & Li, W. CD-HIT: accelerated for clustering the next-generation sequencing data. *Bioinformatics* **28**, 3150–3152 (2012). 23060610 10.1093/bioinformatics/bts565 PMC3516142

[54] Langmead, B. & Salzberg, S. L. Fast gapped-read alignment with Bowtie 2. *Nat. Methods* **9**, 357–359 (2012). 22388286 10.1038/nmeth.1923 PMC3322381

[55] Schubert, M., Lindgreen, S. & Orlando, L. AdapterRemoval v2: rapid adapter trimming, identification, and read merging. *BMC Res. Notes* **9**, 88 (2016). 26868221 10.1186/s13104-016-1900-2 PMC4751634

[56] Li, H. & Durbin, R. Fast and accurate short read alignment with Burrows-Wheeler transform. *Bioinformatics* **25**, 1754–1760 (2009). 19451168 10.1093/bioinformatics/btp324 PMC2705234

[57] Cantalapiedra, C. P., Hernandez-Plaza, A., Letunic, I., Bork, P. & Huerta-Cepas, J. eggNOG-mapper v2: functional annotation, orthology assignments, and domain prediction at the metagenomic scale. *Mol. Biol. Evol.* **38**, 5825–5829 (2021). 34597405 10.1093/molbev/msab293 PMC8662613

[58] Huerta-Cepas, J. et al. eggNOG 5.0: a hierarchical, functionally and phylogenetically annotated orthology resource based on 5090 organisms and 2502 viruses. *Nucleic Acids Res.* **47**, D309–D314 (2019). 30418610 10.1093/nar/gky1085 PMC6324079

[59] Aramaki, T. et al. KofamKOALA: KEGG Ortholog assignment based on profile HMM and adaptive score threshold. *Bioinformatics* **36**, 2251–2252 (2020). 31742321 10.1093/bioinformatics/btz859 PMC7141845

[60] Kanehisa, M. & Goto, S. KEGG: Kyoto Encyclopedia of Genes and Genomes. *Nucleic Acids Res.* **28**, 27–30 (2000). 10592173 10.1093/nar/28.1.27 PMC102409

[61] Brumpton, B. M. et al. The HUNT study: a population-based cohort for genetic research. *Cell Genom.* **2**, 100193 (2022). 36777998 10.1016/j.xgen.2022.100193 PMC9903730

[62] Mbatchou, J. et al. Computationally efficient whole-genome regression for quantitative and binary traits. *Nat. Genet.* **53**, 1097–1103 (2021). 34017140 10.1038/s41588-021-00870-7

[63] Willer, C. J., Li, Y. & Abecasis, G. R. METAL: fast and efficient meta-analysis of genomewide association scans. *Bioinformatics* **26**, 2190–2191 (2010). 20616382 10.1093/bioinformatics/btq340 PMC2922887

[64] Lukina, P. et al. Coeliac disease in the Trondelag Health Study (HUNT), Norway, a population-based cohort of coeliac disease patients. *BMJ Open* **14**, e077131 (2024). 38195172 10.1136/bmjopen-2023-077131 PMC10806793

[65] Yang, J. et al. Common SNPs explain a large proportion of the heritability for human height. *Nat. Genet.* **42**, 565–569 (2010). 20562875 10.1038/ng.608 PMC3232052

[66] Yang, J., Lee, S. H., Goddard, M. E. & Visscher, P. M. GCTA: a tool for genome-wide complex trait analysis. *Am. J. Hum. Genet.* **88**, 76–82 (2011). 21167468 10.1016/j.ajhg.2010.11.011 PMC3014363

[67] Manichaikul, A. et al. Robust relationship inference in genome-wide association studies. *Bioinformatics* **26**, 2867–2873 (2010). 20926424 10.1093/bioinformatics/btq559 PMC3025716

[68] Bulik-Sullivan, B. K. et al. LD Score regression distinguishes confounding from polygenicity in genome-wide association studies. *Nat. Genet.* **47**, 291–295 (2015). 25642630 10.1038/ng.3211 PMC4495769

[69] Trynka, G. et al. Dense genotyping identifies and localizes multiple common and rare variant association signals in celiac disease. *Nat. Genet.* **43**, 1193–1201 (2011). 22057235 10.1038/ng.998 PMC3242065

[70] Zheng, T. et al. Genome-wide analysis of 944 133 individuals provides insights into the etiology of haemorrhoidal disease. *Gut* **70**, 1538–1549 (2021). 33888516 10.1136/gutjnl-2020-323868 PMC8292596

[71] Donertas, H. M., Fabian, D. K., Valenzuela, M. F., Partridge, L. & Thornton, J. M. Common genetic associations between age-related diseases. *Nat. Aging* **1**, 400–412 (2021). 33959723 10.1038/s43587-021-00051-5 PMC7610725

[72] Yengo, L. et al. Meta-analysis of genome-wide association studies for height and body mass index in approximately 700000 individuals of European ancestry. *Hum. Mol. Genet.* **27**, 3641–3649 (2018). 30124842 10.1093/hmg/ddy271 PMC6488973

[73] Machiela, M. J. & Chanock, S. J. LDlink: a web-based application for exploring population-specific haplotype structure and linking correlated alleles of possible functional variants. *Bioinformatics* **31**, 3555–3557 (2015). 26139635 10.1093/bioinformatics/btv402 PMC4626747

[74] Bowden, J., Davey Smith, G. & Burgess, S. Mendelian randomization with invalid instruments: effect estimation and bias detection through Egger regression. *Int. J. Epidemiol.* **44**, 512–525 (2015). 26050253 10.1093/ije/dyv080 PMC4469799

[75] Yavorska, O. O. & Burgess, S. MendelianRandomization: an R package for performing Mendelian randomization analyses using summarized data. *Int. J. Epidemiol.* **46**, 1734–1739 (2017). 28398548 10.1093/ije/dyx034 PMC5510723

[76] Giambartolomei, C. et al. Bayesian test for colocalisation between pairs of genetic association studies using summary statistics. *PLoS Genet.* **10**, e1004383 (2014). 24830394 10.1371/journal.pgen.1004383 PMC4022491

[77] Dekkers, K. F. et al. An online atlas of human plasma metabolite signatures of gut microbiome composition. *Nat. Commun.* **13**, 5370 (2022). 36151114 10.1038/s41467-022-33050-0 PMC9508139

