# Supplementary material for: The HUNT study identifies host genetic factors reproducibly associated with human gut microbiota composition
Source: Nat Genet. 2026 Feb 13;58(3):530–9. doi: 10.1038/s41588-026-02502-4 (PMC12987729; doi:10.1038/s41588-026-02502-4)
Supplement: Supplementary file 1 — Supplementary Note and Supplementary Figs. 1–8. [file 41588_2026_2502_MOESM1_ESM.pdf]

nature genetics

Supplementary information https://doi.org/10.1038/s41588-026-02502-4

The HUNT study identifies host genetic factors reproducibly associated with human gut microbiota composition

In the format provided by the authors and unedited

Supplementary Note

Results - GWAS of gut microbiota species

There was no evidence for test-statistic inflation for any gut microbiota species (median genomic lambda 0.995, range 0.972-1.025, Supplementary Fig. 1 and S2). Conditional analyses did not reveal multiple independent SNP-species signals within the same locus for any species. Using the less stringent GWAS significance threshold ($P < 5.0 \times 10^{-8}$), 79 independent loci were identified, including 106 unique SNP-species associations (Supplementary Table 6).

In sensitivity analyses, we excluded participants prescribed antibiotic treatment within the last three months or adjusted for antibiotic use, revealing essentially unchanged effect estimates for the SNP-species associations (Supplementary Table 8).

Sensitivity analyses using logistic regression (species presence or absence) for the species with a prevalence between 30% and 50% and having replicated significant SNP-species associations yielded similar results (Supplementary Table 9).

Bristol stool scale, a measure of bowel motility, was associated with the relative abundances of some, but not all, of the twelve replicated species (Supplementary Table 9). However, the associations for the twelve identified replicated SNP-species associations remained essentially unchanged after adjustment for Bristol stool scale (Supplementary Table 9).

We also performed sensitivity analyses for the twelve top replicated findings using the alternative, frequently used software, BOLT-LM[1], which uses a genetic relationship matrix to correct for population structure and relatedness. BOLT-LMM analyses revealed essentially similar results as when using REGENIE (Supplementary Table 9). For the twelve replicated SNP-species signals, we also conducted sensitivity analyses, restricting to 8,221 unrelated subjects and adjusting for ten principal components, with similar results (Supplementary Table 9). Finally, we performed sensitivity analyses excluding cohabitation, with similar results (Supplementary Table 9).

Sensitivity analyses using the centred log-ratio (CLR) transformation yielded similar associations for all replicated SNP-species associations except for the association with *Mediterraneibacter*

faecis at the *FUT2* locus. However, the three other signals at the *FUT2* locus were replicated using CLR transformation and all four SNP-species signals at the *FUT2* locus were replicated in the Swedish replication cohorts (Supplementary Table 9).

Heritability was also estimated using linkage disequilibrium (LD) score regression, showing a heritability between 0 % and 19 % for the 546 evaluated gut microbiota species (Supplementary Table 5). The species heritability estimates obtained using GCTA and LD score regression were correlated (Pearson correlation 0.49, $P = 7.3 \times 10^{-31}$). The heritability for Shannon index and richness using LD Score regression was 10.5 ± 4.0 % and 20.6 ± 3.9 %, respectively.

Results - GWAS of KEGG functionality modules

There was no evidence for test-statistic inflation for any KEGG functionality module (median genomic lambda 0.996, range 0.975-1.018; Supplementary Fig. 5).

Results - The genetic signal at the *HLA-DQB1* locus

Further analyses revealed that the relative abundance of *Agathobacter sp000434275* was moderately associated with the coeliac disease risk allotypes HLA-DQ2.5, HLA-DQ8 and HLA-DQ2.2 (N = 7,105 HLA-DQ2.5, HLA-DQ8 or HLA-DQ2.2 positive and 5,529 negative subjects; OR 0.92; 95 % CI 0.89-0.96 per SD increase in relative abundance of *Agathobacter sp000434275*, $P = 7.3 \times 10^{-5}$). This modest association is also illustrated in a distribution plot (Supplementary Fig. 6B). The association of the HLA-DQ2.5, HLA-DQ8 and HLA-DQ2.2 risk allotypes with *Agathobacter sp000434275* was less pronounced compared with the association of coeliac disease with *Agathobacter sp000434275* (Figure S6), likely because only a minor part of the HLA-DQ2.5, HLA-DQ8 and HLA-DQ2.2 positives had coeliac disease.

Two-sample MR using only one available genome-wide significant genetic instrument ($P < 5 \times 10^{-8}$) indicated that a higher relative abundance of *Agathobacter sp000434275* was causally associated with a reduced risk of coeliac disease. However, no evidence of causality was observed when using a more inclusive *P*-value threshold ($P < 1.0 \times 10^{-6}$, resulting in three genetic instruments located in different loci) for selection of genetic instruments for the

Agathobacter sp000434275 exposure (Supplementary Table 25 and S26). Reverse MR revealed that having coeliac disease was causally associated with reduced relative abundance of *Agathobacter sp000434275* (beta = -0.036, standard error (SE) = 0.014, $P = 9.9 \times 10^{-3}$, Supplementary Table 25 and S26). Thus, there is some evidence that coeliac disease reduces the relative abundance of *Agathobacter sp000434275*, while the possible impact of *Agathobacter sp000434275* on coeliac disease is unclear.

We also observed that the relative abundance of *Agathobacter sp000434275* was positively associated with circulating levels of the gut microbiota-derived metabolite *3-phenylpropionate* (top metabolite association for *Agathobacter sp000434275*, $P = 9{,}0 \times 10^{-64}$, Spearman correlation ($r_s$) = 0.18, N = 8,582, Supplementary Table 27).

Results - The genetic signals at the *MUC12* locus

Two-sample MR using only one available genome-wide significant genetic instrument ($P < 5 \times 10^{-8}$) indicated that a higher relative abundance of *Coprobacillus cateniformis* was associated with decreased risk of haemorrhoidal disease. When using a more inclusive *P*-value threshold ($P < 1.0 \times 10^{-6}$, resulting in four genetic instruments located in different loci) for selection of genetic instruments, we observed some evidence of causality (Supplementary Table 25 and S26). However, leave-one-out cross-validation of this MR analysis revealed that the association was mainly driven by the study-wide significant signal rs4556017 for *Coprobacillus cateniformis*. Reverse MR revealed no evidence for a causal effect of haemorrhoidal disease on *Coprobacillus cateniformis* (Supplementary Table 25 and S26).

Results - Sex and age interaction analyses

We did not observe significant sex or age interactions for the top SNP-species associations in the six replicated loci (adjustment for six SNPs and two interactions tested, $P > 4.0 \times 10^{-3}$, Supplementary Table 23).

Results - Interaction between genetic variants at the *ABO* and *FUT2* loci

Next, we determined if any of the ABO blood groups displayed a significant interaction with FUT2 secretor status for the relative abundance of *Mediterraneibacter torques*. We observed that there was a significant interaction between the sum of the number of A and B alleles and FUT2 secretor status for the relative abundance of *Mediterraneibacter torques* (Supplementary Table 30).

Two-sample MR using only one available genome-wide significant genetic instrument ($P < 5 \times 10^{-8}$) indicated that a higher relative abundance of *Clostridium sp900540255* was causally associated with a modestly reduced risk of high cholesterol but no evidence of causality was observed when using a more inclusive *P*-value threshold ($P < 1.0 \times 10^{-6}$, resulting in five genetic instruments located in different loci) for selection of genetic instruments for the *Clostridium sp900540255* exposure (Supplementary Table 25, and S26). Reverse MR revealed no evidence for a causal effect of high cholesterol on relative abundance of *Clostridium sp900540255* (Supplementary Table 25 and S26).

Results - Evidence of an effect of BMI on gut microbiota composition

Analyses restricted to the 98 gut microbiota species (Supplementary Table 33) with available genetic instruments (selected genetic variants had $P < 5 \times 10^{-8}$, F-statistic > 12, Supplementary Table 25) derived from the current GWAS study showed no correlation between the betas of the observational associations and the betas for the causal effect of the different gut microbiota species on BMI (Pearson correlation -0.15, $P = 0.13$, Supplementary Table 33). However, there was some evidence that seven gut microbiota species were causally associated with either decreased (six species) or increased (one species) BMI (Bonferroni adjusted significance of $P < 5.0 \times 10^{-4}$; adjusting for 98 MR analyses; Supplementary Table 33).

Discussion - Findings in cladogram

As visualised in the cladogram (Fig 2), there are some overlaps between the species significantly associated with the top signals in the *ABO* and *FUT2* loci. At the same time, we observe more

distinct associations with the four other replicated loci (Fig 2), suggesting different etiology for these host microbiota interactions.

Discussion – The *LCT* locus and KEGG functionality modules

One may speculate that the observed association of higher relative abundance of *Bifidobacterium adolescentis* and the *Glutamate transport system* could be due to the efficient glutamate to gamma-aminobutyric acid (GABA) conversion in *Bifidobacterium adolescentis* strains.[2] This notion is supported by our finding that the relative abundance of *Bifidobacterium adolescentis* explained as much as 61 % of the variance in the KEGG *Glutamate transport system* module, but further studies are required to validate this hypothesis.

Unexpectedly, we observed an association between the index SNP rs182549-T allele in *LCT* and *Crassulacean acid metabolism*. As *crassulacean acid metabolism* is only reported in plants and not in the bacteria of the gut[3], this association seems to originate either from misclassification of this KEGG functionality module or from dietary influences in certain individuals with lactose intolerance.

Discussion - *Agathobacter sp000434275* and 3-phenylpropionate

A high relative abundance of *Agathobacter sp000434275* was associated with high circulating levels of the gut microbiota derived metabolite 3-phenylpropionate, which could indicate a high fibre intake and is associated with healthy properties such as reduced risk of type 2 diabetes.[4] A high-fibre diet is thought to induce a metabolic transition from mucus-degrading to fibre-degrading activities in the colon, as carbohydrates from fibre are more easily metabolized by the gut microbiota.[5] Patients with coeliac disease have reduced fibre intake[6] and reduced abundance of *Agathobacter sp000434275*. We speculate that the positive association between *Agathobacter sp000434275* and 3-phenylpropionate could result from the reduced fibre intake in coeliac disease patients. However, these findings should be validated in external data which provide detailed dietary information.

Discussion - *ACHE* as an alternative gene for *Coprobacillus cateniformis*

A previous study suggested *ACHE* as candidate gene for hemorrhoidal disease in this locus,[7] describing that the top SNP rs4556017 for hemorrhoidal disease has eQTLs for *ACHE*. However, no colocalization evidence was provided in any tissue and no eQTL was presented in the gastrointestinal tract. The eQTLs of this signal are, according to the Open Targets platform, associated with *ACHE* expression in arteries, thyroid and tibial nerves. Although we believe that *MUC12* is a more plausible candidate gene for the observed *Coprobacillus cateniformis* association, we cannot exclude a role of *ACHE* in arteries.

Discussion - *FUT2* and cardiovascular parameters

The index SNP in the *FUT2* locus was also associated with a composite cardiovascular-related parameter. Further separate analyses revealed that an association with high cholesterol and hypertension mainly drove this association. A connection between *Clostridium sp900540255* and high cholesterol was supported by strong evidence for genetic colocalization. However, it is possible that the lack of BMI as a covariate in the statistical models may have influenced these associations. Future studies are needed to investigate the possible causality between *Clostridium sp900540255* and high cholesterol and/or hypertension.

Discussion - BMI and gut microbiota composition

There was some evidence that seven species were causally associated with BMI. Still, these MR results may have been biased by weak instruments or horizontal pleiotropy, such as for the two species with genetic instruments in the *LCT* locus,[8-10] thereby violating key MR assumptions.

Further meta-analyses of the causal effect of BMI on gut microbiota composition using other cohorts besides HUNT should validate the findings in the present study. In addition, a limitation in the present study is the lack of dietary information for adjustment in the models determining the causality between BMI and gut microbiota.

Methods - Discovery cohort - HUNT

Data and biological samples have been collected through four cross-sectional surveys, from 1984-2019 (HUNT1-4). About 230,000 participants (aged ≥ 20 years) have participated in at least one survey, of which almost 95,000 participants have submitted one or more biological samples. Of these, approximately 88,000 HUNT participants (HUNT2-4) have been genotyped.[11]

Study participants were asked to score their stool sample according to the Bristol stool scale.[12] This scale is numbered 1–7, with one being the hardest type of stool and seven being completely liquid. For statistical analyses, the Bristol stool scale data was recoded into 3 categories: Bristol stool scale 1–2 was categorized as hard stool and coded as 1, Bristol stool scale 3–4 were categorized as normal stool and coded as 2, and Bristol stool scale 5–7 were categorized as loose stool and coded as 3.[13] For sensitivity analyses excluding cohabitation between individuals, we only selected one participant per postal address at the time of invitation to HUNT4, and excluded participants with unknown postal address.

Methods – DNA isolation of HUNT samples:

Stool collection and DNA isolation and quantification have been performed using a standardized procedure, as previously described[13] prior to sequencing and microbiome profiling at Clinical Microbiomics in Denmark (Fig 1A, Supplementary Note). [14] In brief, stool samples were collected on filter papers in the homes of the participants and then sent to the HUNT Biobank for storage at –80°C. Three 6 mm discs were punched out from each filter card into an allocated well on MagMAX™ 96 Deep Well Plates (Thermo Fisher Scientific, Waltham, USA). After bead-beating to disrupt microbial cell walls, DNA was isolated using the Microbiome MagMAX Ultra kit (Thermo Fisher Scientific) following the manufacturer's recommendations on KingFisher™ Flex (Thermo Fisher Scientific). The amount of DNA was quantified using Quant-iT™ PicoGreen™ dsDNA Reagent (Thermo Fisher Scientific).

Methods - Limit of detection of species in HUNT

We have calculated the limit of detection (LOD; the relative abundance at which we have 95 % chance of detecting a species) based on the number of reads that map the CHAMP signature genes (used for profiling and abundance calculations as described in *Pita et al*)[14] and the profiling algorithm's species sensitivity (requiring at least five signatures read counts). This established that the average species LOD among the HUNT participants was 0.0009 %.

Methods - Genotyping and imputation - HUNT

More than 88,000 HUNT participants have been genotyped using one of four Illumina HumanCoreExome arrays: 12 v.1.0, 12 v.1.1, UM HUNT Biobank v1.0, and UM HUNT Biobank v2.0.[11] Genotype calling was performed with GenTrain v.2.0 in GenomeStudio v.2011.1 (Illumina). Samples were excluded if they had call rate < 99 %, large chromosomal copy number variants, contamination > 2.5 % as estimated with BAF Regress,[15] or a discrepancy between genetically inferred sex and reported gender. Genetic variants were excluded if they deviated from Hardy-Weinberg Equilibrium ($P < 10^{-4}$). Samples were phased with Eagle2 v.2.0.5 (https://alkesgroup.broadinstitute.org/Eagle/). All variants were imputed from the Haplotype Reference Consortium (HRC) v1.1 reference panel with the positional Burrows-Weeler transform (PBWT) v3.1 https://github.com/richarddurbin/pbwt).[16] ABO blood groups were determined using four genetic variants as proposed by *Paré et al* (with rs507666, rs687289, rs8176746 and rs8176704 as proxies for the alleles A1, O, B and A2, respectively).[17] HLA-DQ2.5, HLA-DQ2.2, and HLA-DQ8 risk allotypes were determined from alleles imputed using the T1DGC HLA Reference Panel[18] and Cook HLA software.[19] The imputed data were validated against genotyped allotypes for a subset of 2,189 HUNT4 participants. The overall accuracy for identifying carriers of HLA-DQ2.5, HLA-DQ2.2, or HLA-DQ8 versus any other HLA-DQ allotype was estimated at 0.93.

Methods - Colocalization

To assess if any of the identified gut microbiota species loci were consistent with having shared causal variants with selected human diseases or tissue-specific eQTLs of interest, we combined their GWAS summary statistics and performed a Bayesian colocalization analysis as implemented in the R package coloc.[20] We included diseases that were strongly associated ($P < 1.0 \times 10^{-17}$) with the identified index SNPs in any of the identified novel loci (*HLA-DQB1, MUC12, SLC37A2, FUT2*) using the PheWAS tool available in the Open Targets platform (https://platform.opentargets.org/), as well as the gene identified as the most likely causal gene by the Open Targets platform and having an eQTL in a biologically plausible tissue. The variance in each trait (inverse rank transformed relative abundances of gut microbiota species, diseases, or eQTLs) was estimated from the sample sizes and minor allele frequencies. We set the prior probability of a genetic variant being associated with only the gut microbiota species, only the disease/eQTL or both traits to be 10^{-4}, 10^{-4}, and 10^{-6} respectively. We considered posterior probabilities above 80 % to give strong support for a common causal variant for the two traits.

References to Supplementary Note

1. Loh, P.R., *et al.* Efficient Bayesian mixed-model analysis increases association power in large cohorts. *Nat Genet* **47**, 284-290 (2015).
2. Duranti, S., *et al.* Bifidobacterium adolescentis as a key member of the human gut microbiota in the production of GABA. *Sci Rep* **10**, 14112 (2020).
3. Gilman, I.S. & Edwards, E.J. Crassulacean acid metabolism. *Current Biology* **30**, R57-R62 (2020).
4. Wang, Z., *et al.* Gut Microbiota and Blood Metabolites Related to Fiber Intake and Type 2 Diabetes. *Circ Res* **134**, 842-854 (2024).
5. Desai, M.S., *et al.* A Dietary Fiber-Deprived Gut Microbiota Degrades the Colonic Mucus Barrier and Enhances Pathogen Susceptibility. *Cell* **167**, 1339-1353 e1321 (2016).
6. Laurikka, P., *et al.* Dietary Factors and Mucosal Immune Response in Celiac Disease Patients Having Persistent Symptoms Despite a Gluten-free Diet. *J Clin Gastroenterol* **53**, 507-513 (2019).
7. Zheng, T., *et al.* Genome-wide analysis of 944 133 individuals provides insights into the etiology of haemorrhoidal disease. *Gut* **70**, 1538-1549 (2021).
8. Kurilshikov, A., *et al.* Large-scale association analyses identify host factors influencing human gut microbiome composition. *Nat Genet* **53**, 156-165 (2021).
9. Lopera-Maya, E.A., *et al.* Effect of host genetics on the gut microbiome in 7,738 participants of the Dutch Microbiome Project. *Nat Genet* **54**, 143-151 (2022).
10. Qin, Y., *et al.* Combined effects of host genetics and diet on human gut microbiota and incident disease in a single population cohort. *Nat Genet* **54**, 134-142 (2022).
11. Brumpton, B.M., *et al.* The HUNT study: A population-based cohort for genetic research. *Cell Genom* **2**, 100193 (2022).

12. O'Donnell, L.J., Virjee, J. & Heaton, K.W. Detection of pseudodiarrhoea by simple clinical assessment of intestinal transit rate. *BMJ* **300**, 439-440 (1990).
13. Grahnemo, L.*, et al.* Cross-sectional associations between the gut microbe Ruminococcus gnavus and features of the metabolic syndrome. *Lancet Diabetes Endocrinol* **10**, 481-483 (2022).
14. Pita, S.*, et al.* CHAMP delivers accurate taxonomic profiles of the prokaryotes, eukaryotes, and bacteriophages in the human microbiome. *Front Microbiol* **15**, 1425489 (2024).
15. Jun, G.*, et al.* Detecting and estimating contamination of human DNA samples in sequencing and array-based genotype data. *Am J Hum Genet* **91**, 839-848 (2012).
16. Durbin, R. Efficient haplotype matching and storage using the positional Burrows-Wheeler transform (PBWT). *Bioinformatics* **30**, 1266-1272 (2014).
17. Pare, G.*, et al.* Novel association of ABO histo-blood group antigen with soluble ICAM-1: results of a genome-wide association study of 6,578 women. *PLoS Genet* **4**, e1000118 (2008).
18. Jia, X.*, et al.* Imputing amino acid polymorphisms in human leukocyte antigens. *PLoS One* **8**, e64683 (2013).
19. Cook, S.*, et al.* Accurate imputation of human leukocyte antigens with CookHLA. *Nat Commun* **12**, 1264 (2021).
20. Giambartolomei, C.*, et al.* Bayesian test for colocalisation between pairs of genetic association studies using summary statistics. *PLoS Genet* **10**, e1004383 (2014).

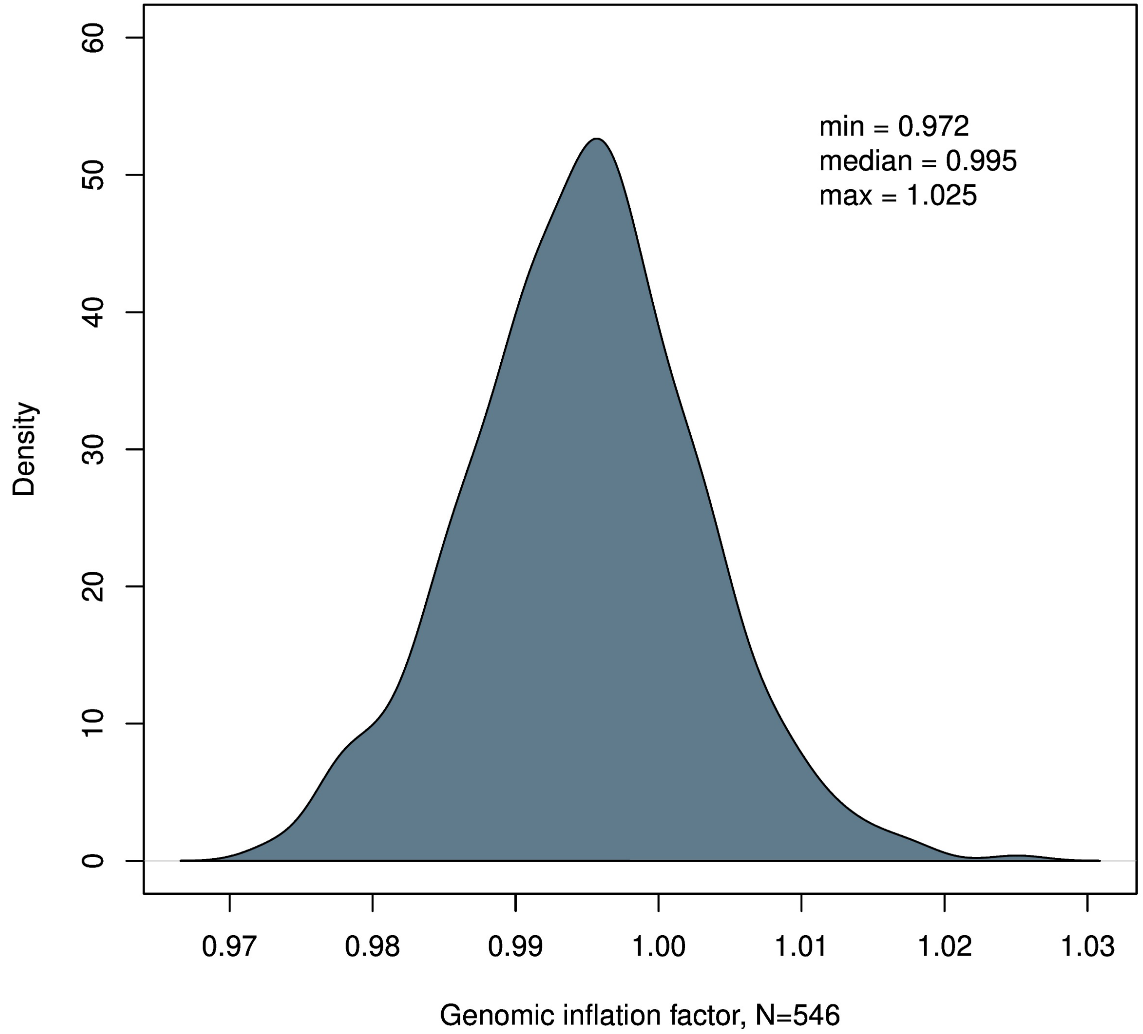

Supplementary Fig. 1 Density plot of the genomic inflation factor for the 546 evaluated species.

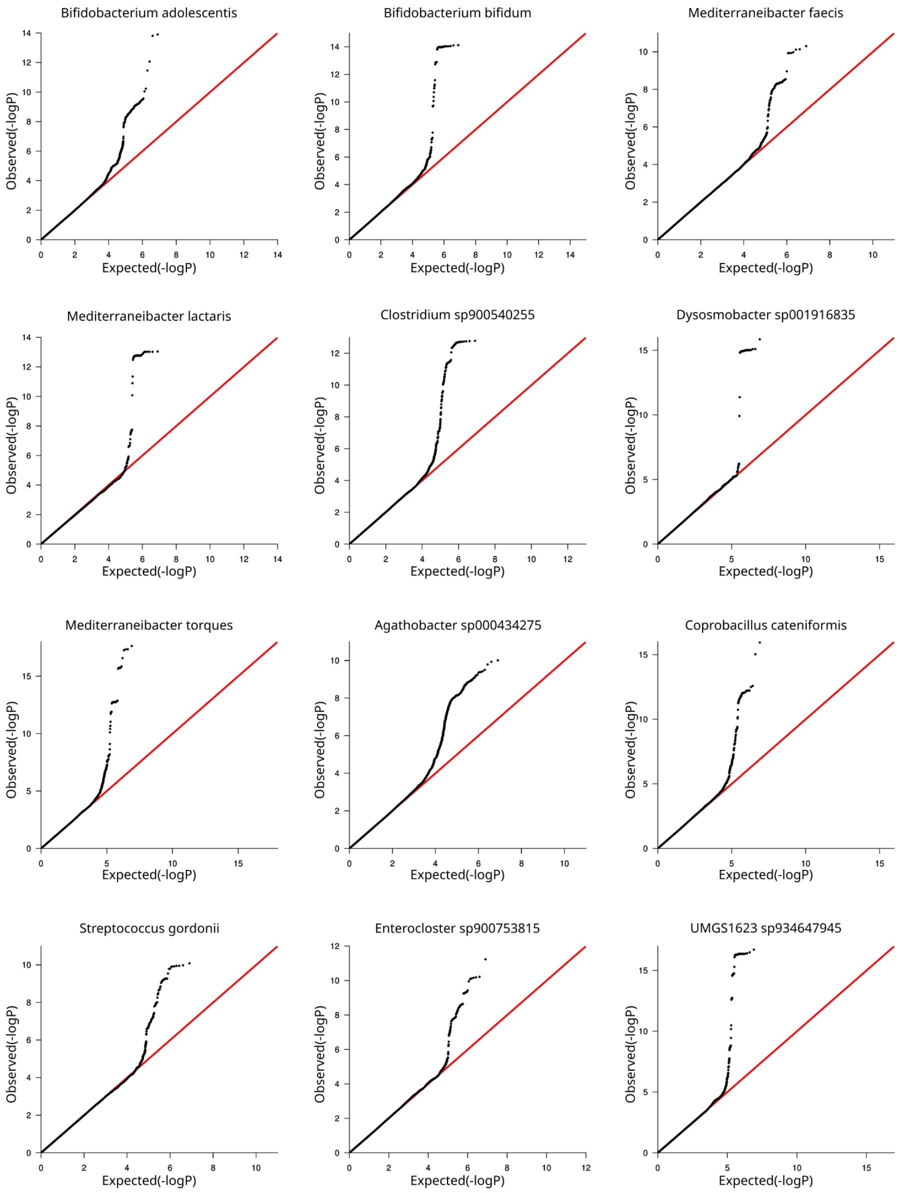

Supplementary Fig. 2 Q/Q plots for the GWASs of the twelve species with replicated SNP-species associations. The observed unadjusted P-values are from two-sided z-tests.

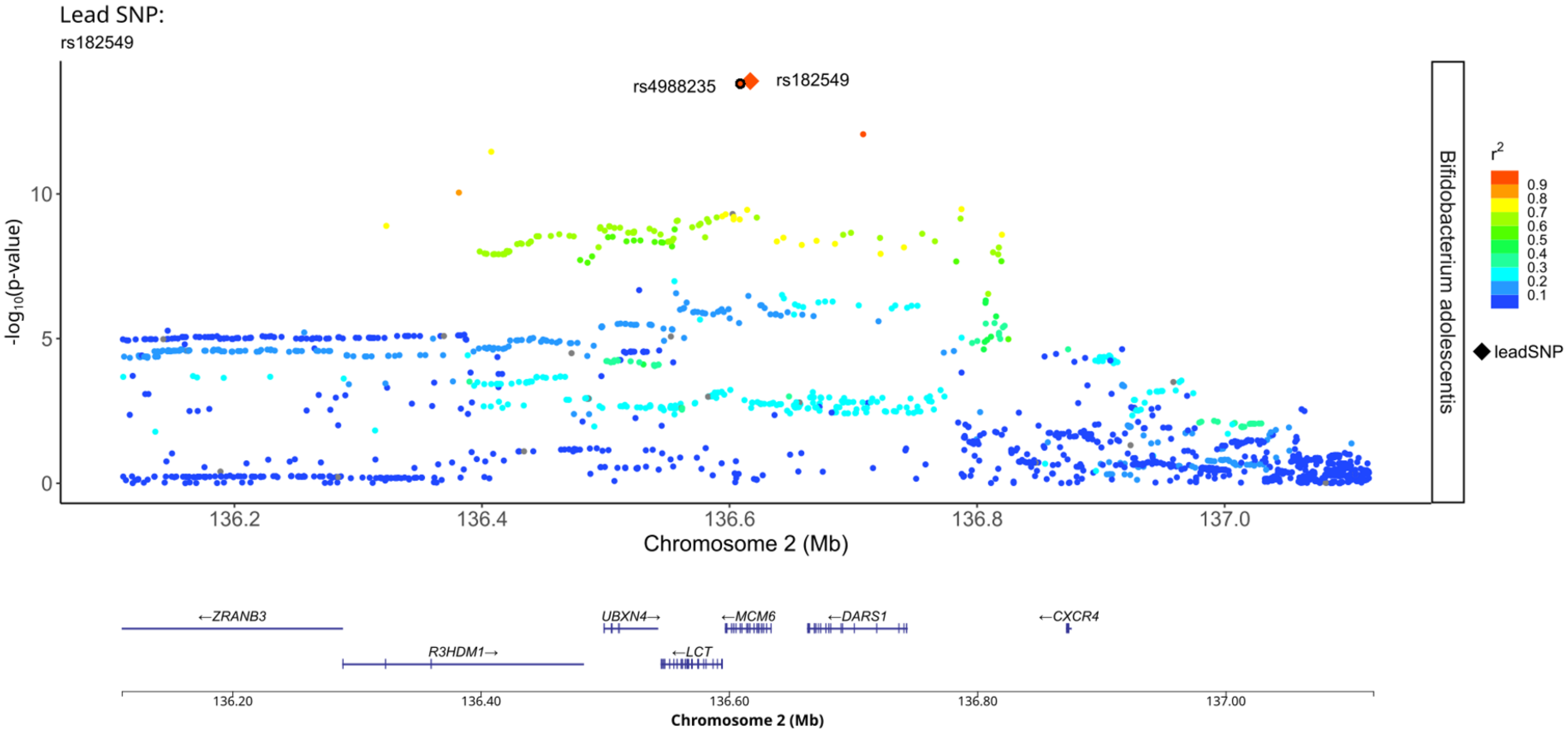

Supplementary Fig. 3a

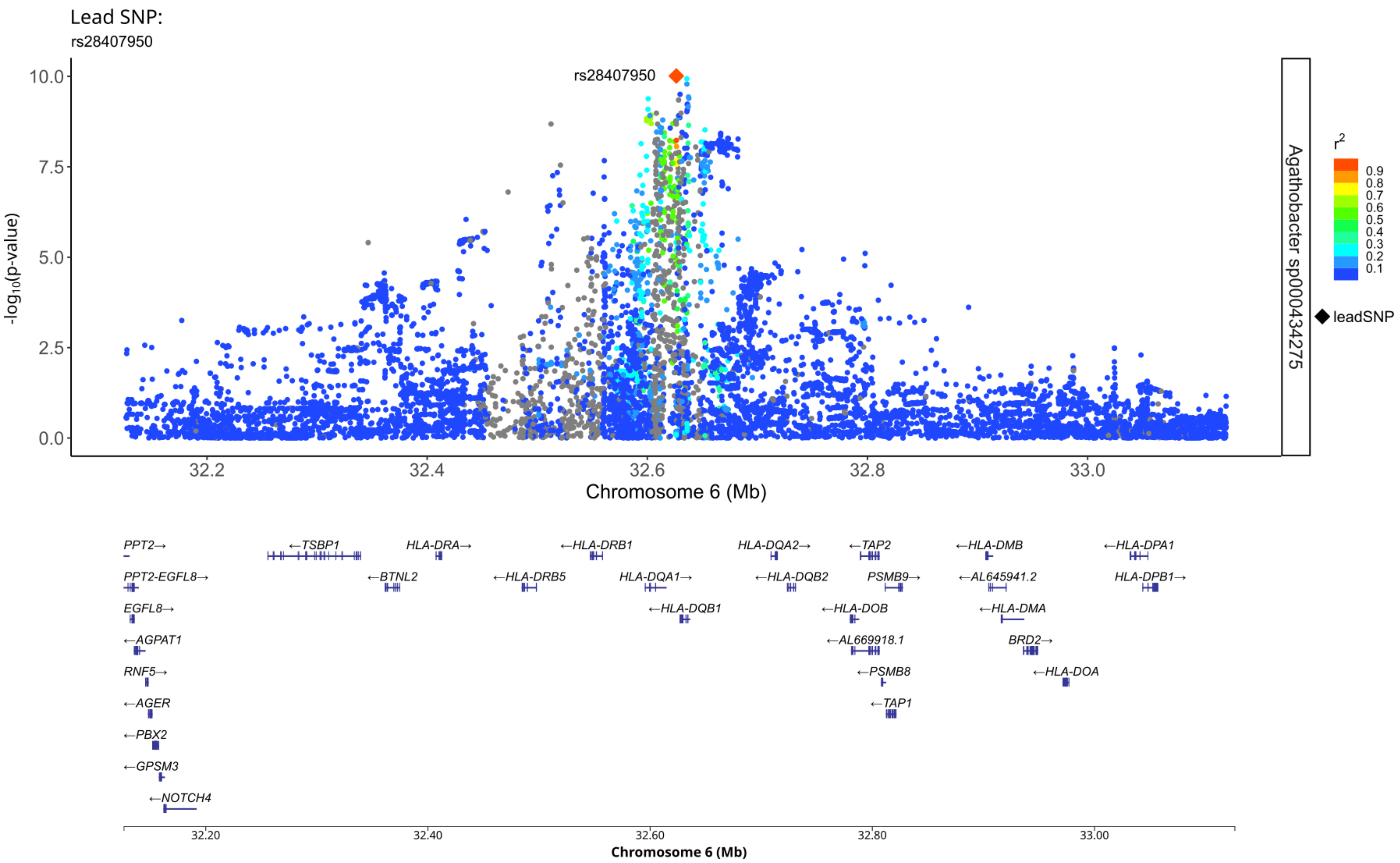

Supplementary Fig. 3b

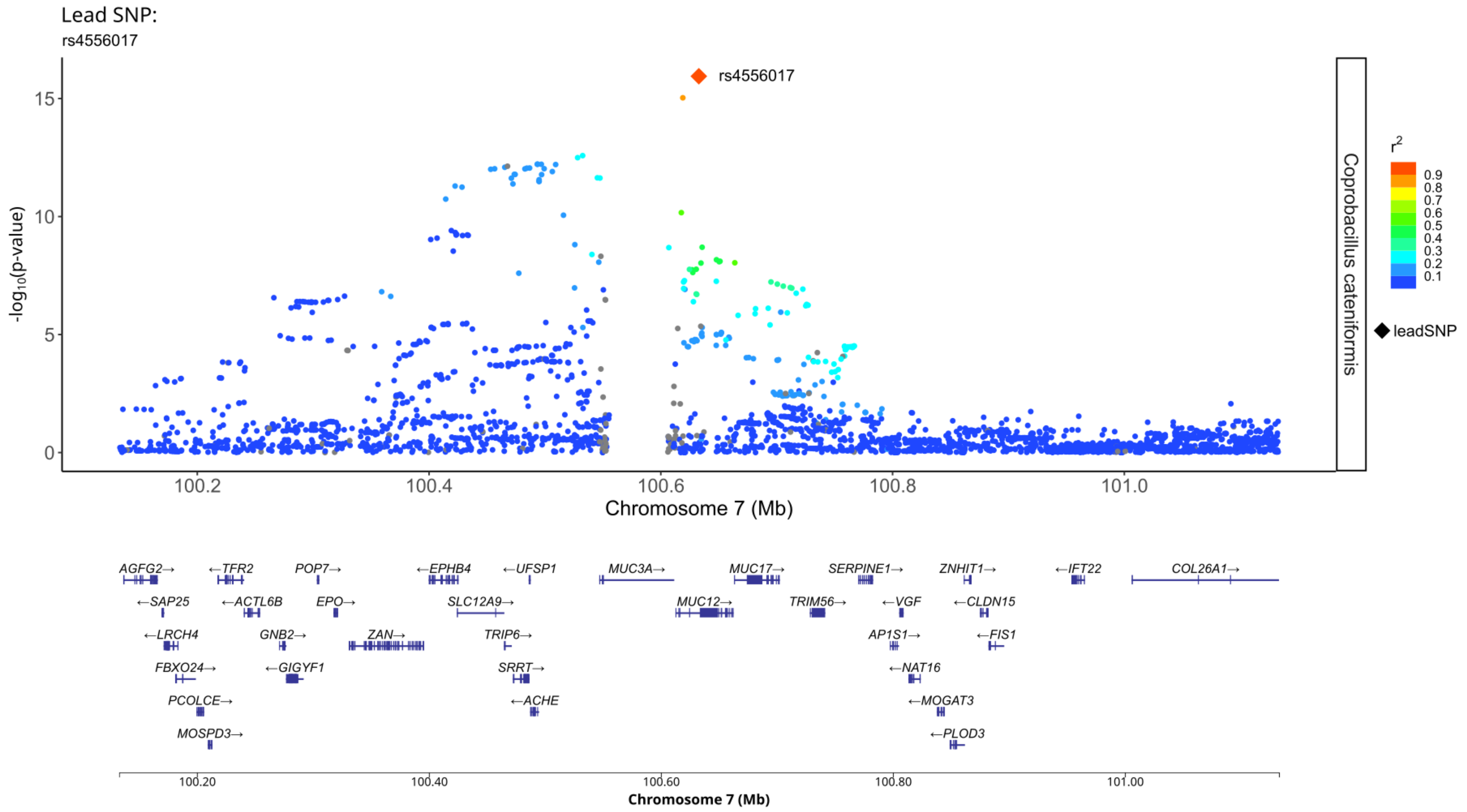

Supplementary Fig. 3c

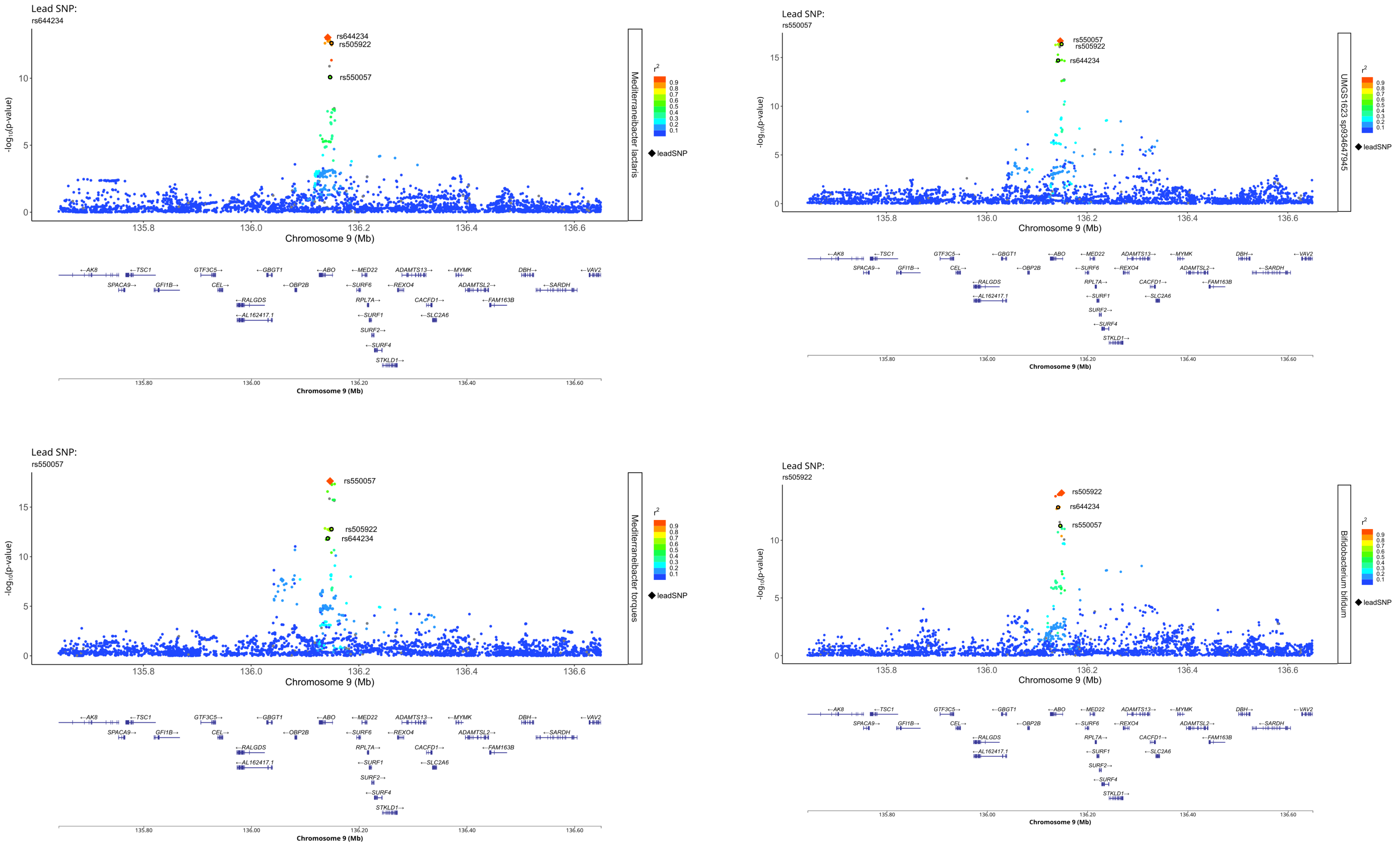

Supplementary Fig. 3d

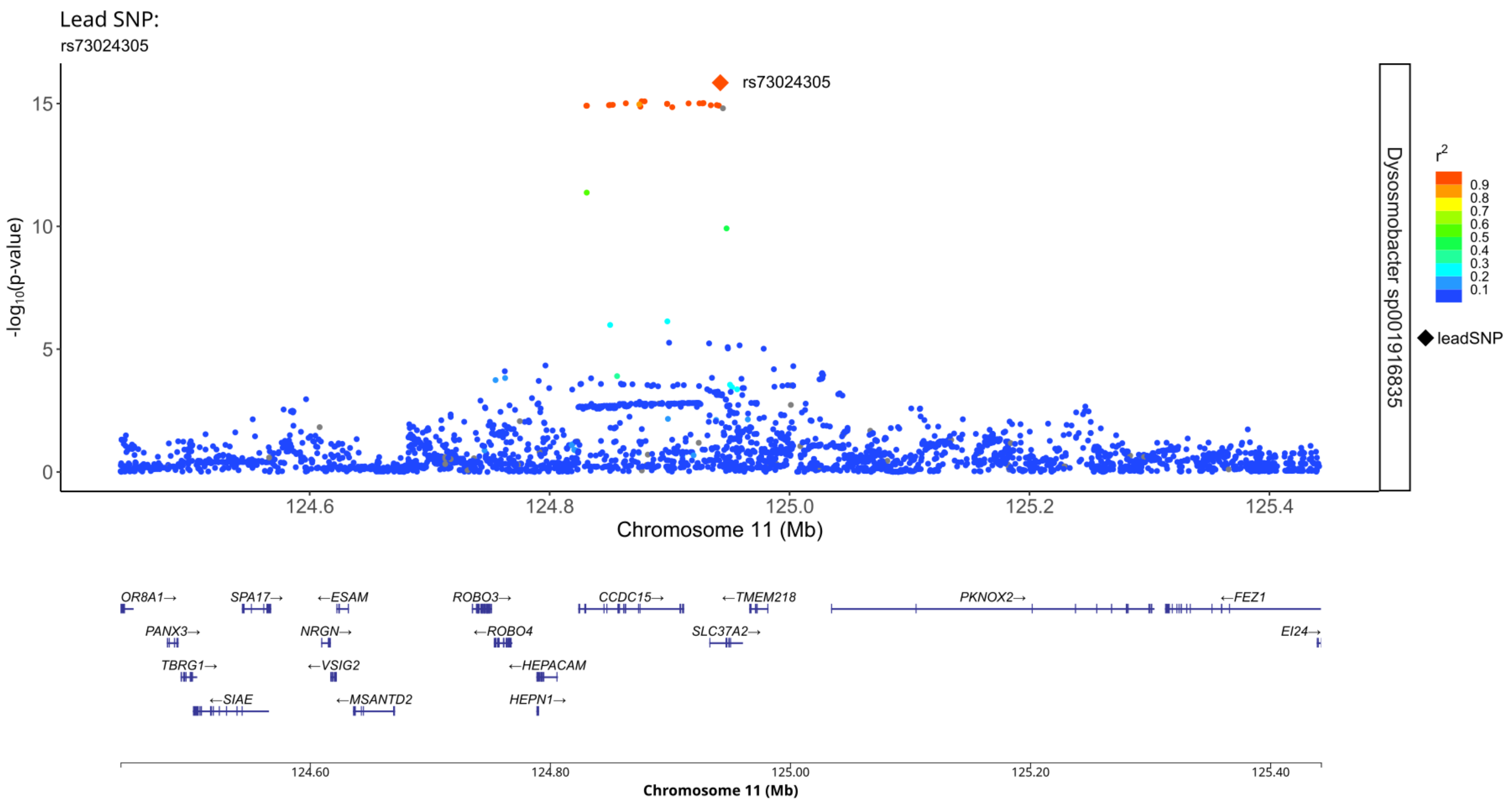

Supplementary Fig. 3e

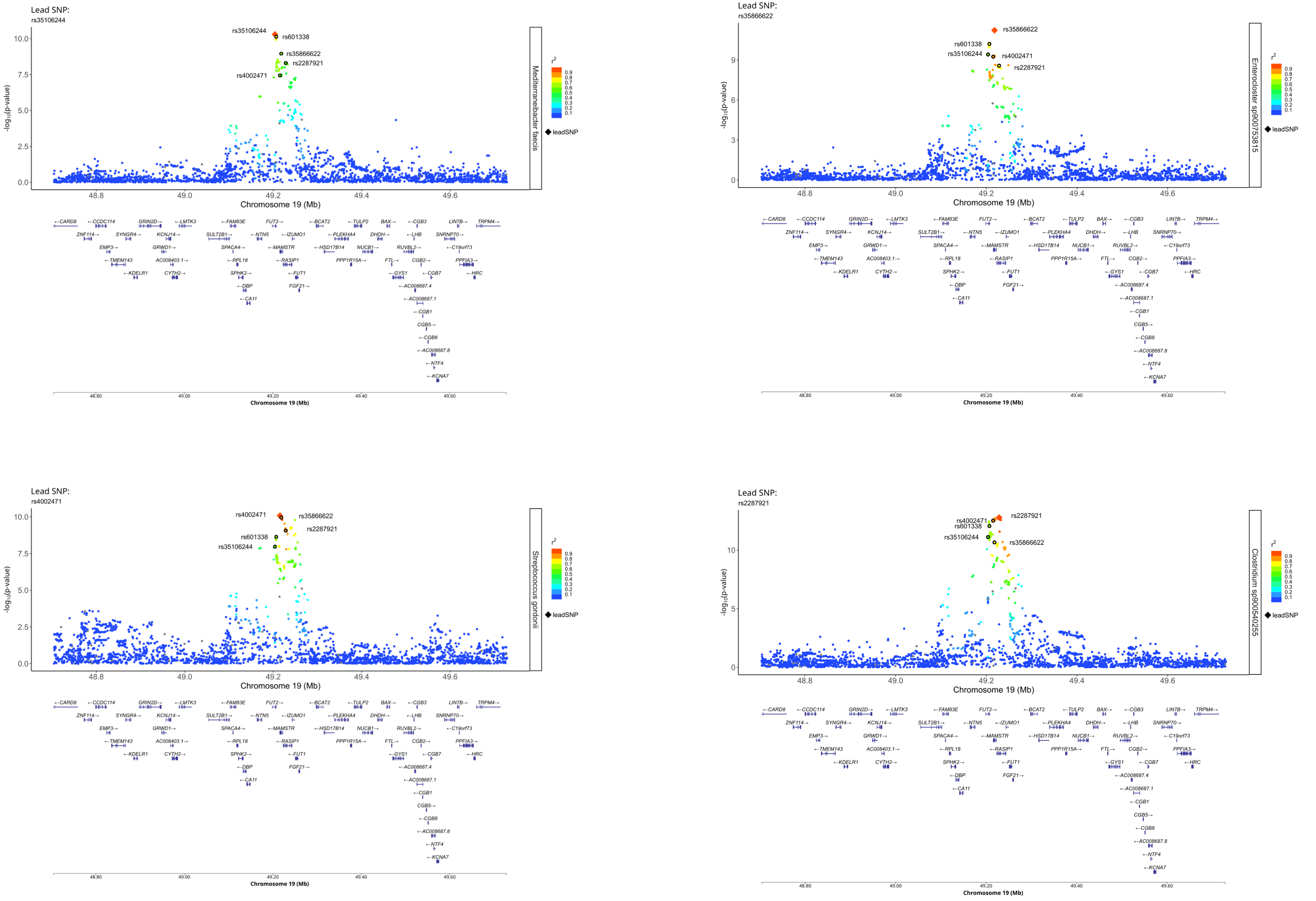

Supplementary Fig. 3f

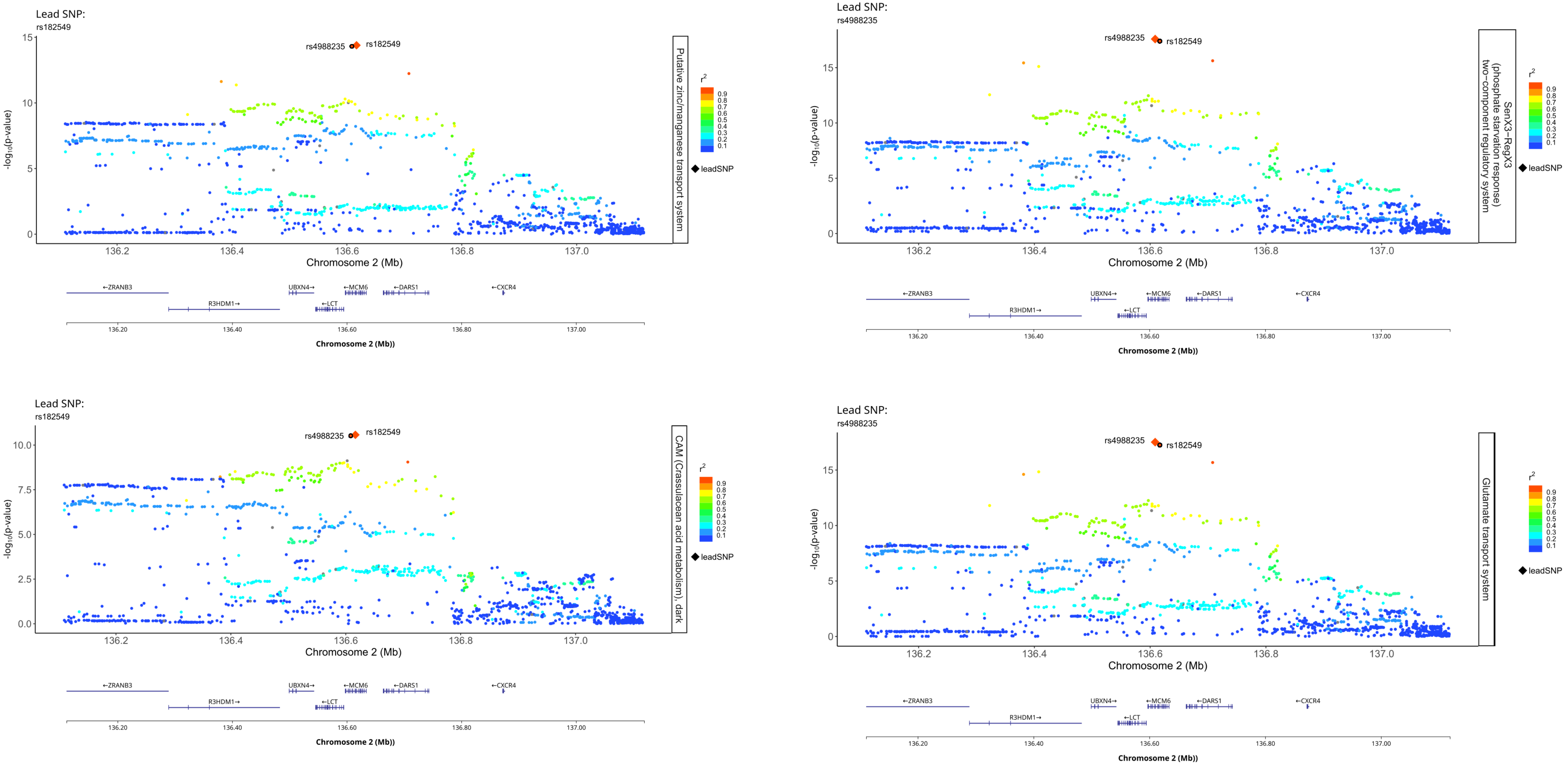

Supplementary Fig. 3g

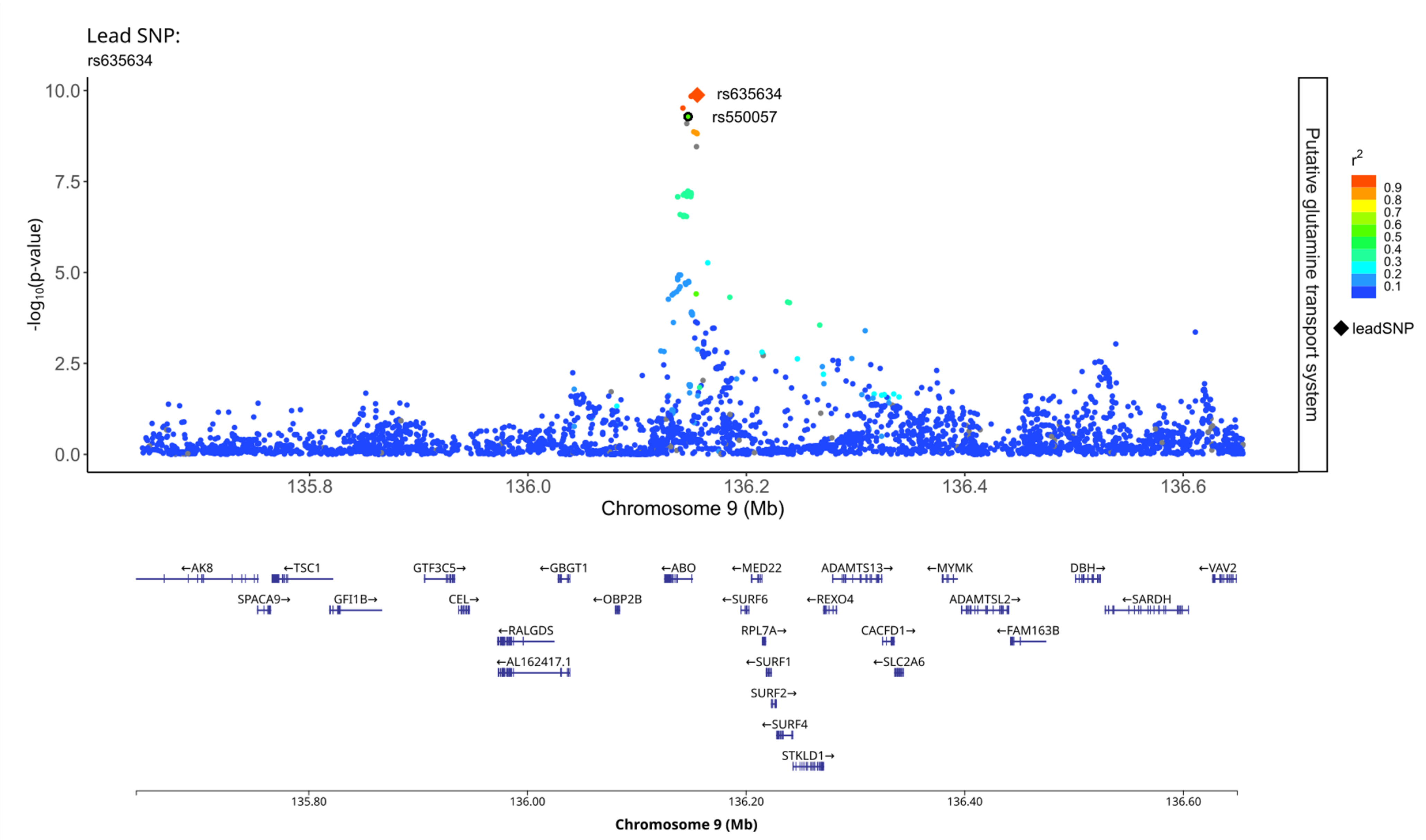

Supplementary Fig. 3h

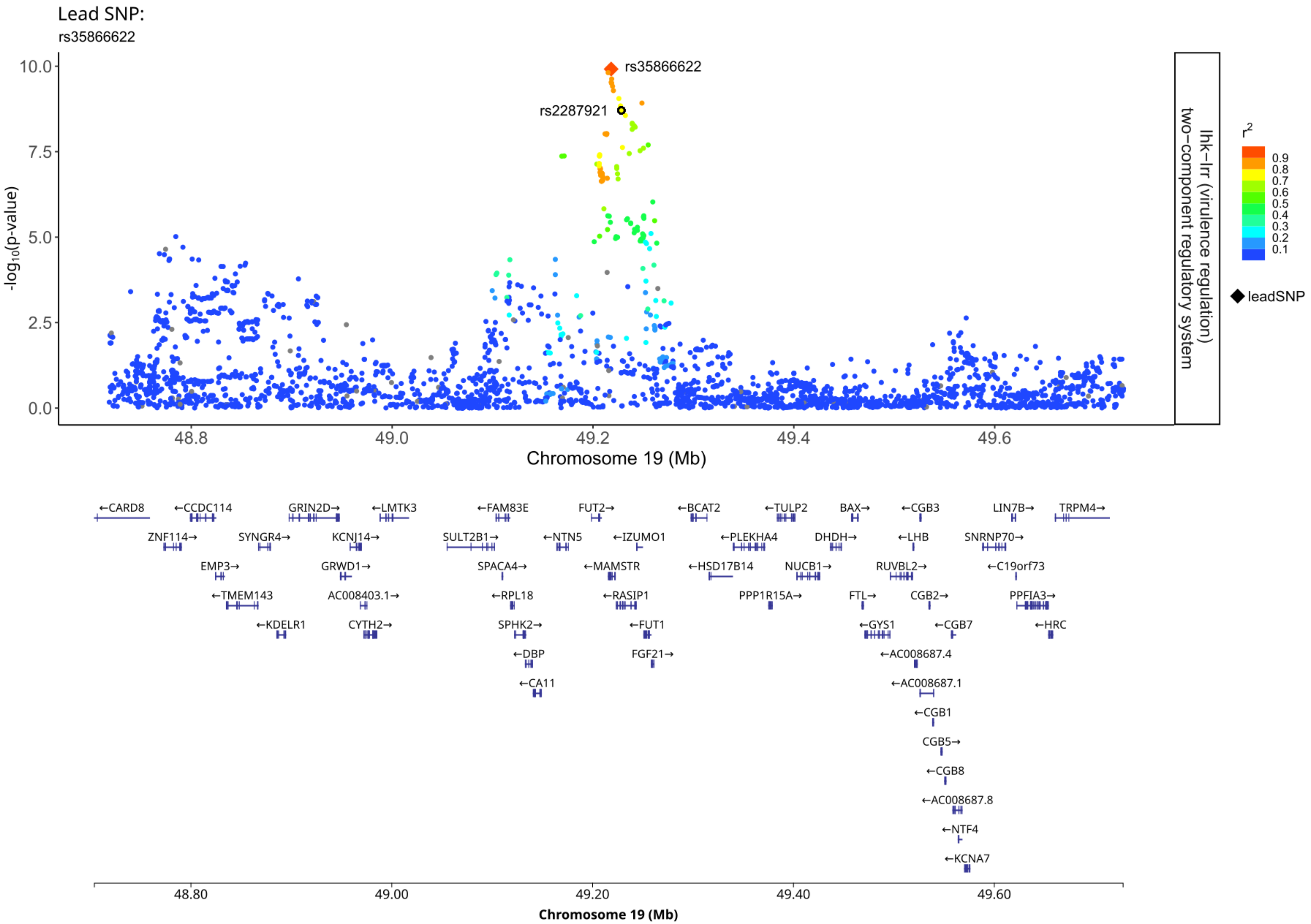

Supplementary Fig. 3i

Supplementary Fig. 3 Regional associations plots of replicated SNP associations with gut microbiota species (a-f) and with KEGG functionality modules (g-i).

a) rs182549 at chr 2 with *Bifidobacterium adolescentis.*

b) rs28407950 at chr 6 with *Agathobacter sp000434275.*

c) rs4556017 at chr 7 with *Coprobacillus cateniformis.*

d) At chr 9; rs644234 with *Mediterraneibacter lactaris* (upper left), rs550057 with *Mediterraneibacter torques* (lower left), rs550057 with *UMGS1623 sp934647945* (upper right), and rs505922 with *Bifidobacterium bifidum* (lower right).

e) rs73024305 at chr 11 with *Dysosmobacter sp001916835.*

f) At chr 19; rs35106244 with *Mediterraneibacter faecis* (upper left), rs4002471 with Streptococcus gordonii (lower left), rs35866622 with *Enterocloster sp900753815* (upper right), rs2287921 with *Clostridium sp900540255* (lower right).

g) At chr 2; rs4988235 with *SenX3-RegX3 (phosphate starvation response) two-component regulatory system* (upper left), rs182549 with *Putative zinc/manganese transport system* (lower left), rs4988235 with *Glutamate transport system* (upper right), and rs182549 with *CAM (Crassulacean acid metabolism), dark* (lower right).

h) rs635634 at chr 9 with *Putative glutamine transport system.*

i) rs35866622 at chr 19 with *Ihk-Irr (virulence regulation) two-component regulatory system.*

a-i) Index SNPs within the same loci but associated with other species or KEGG functionalities are highlighted with black circles.

The observed unadjusted P-values are from two-sided z-tests.

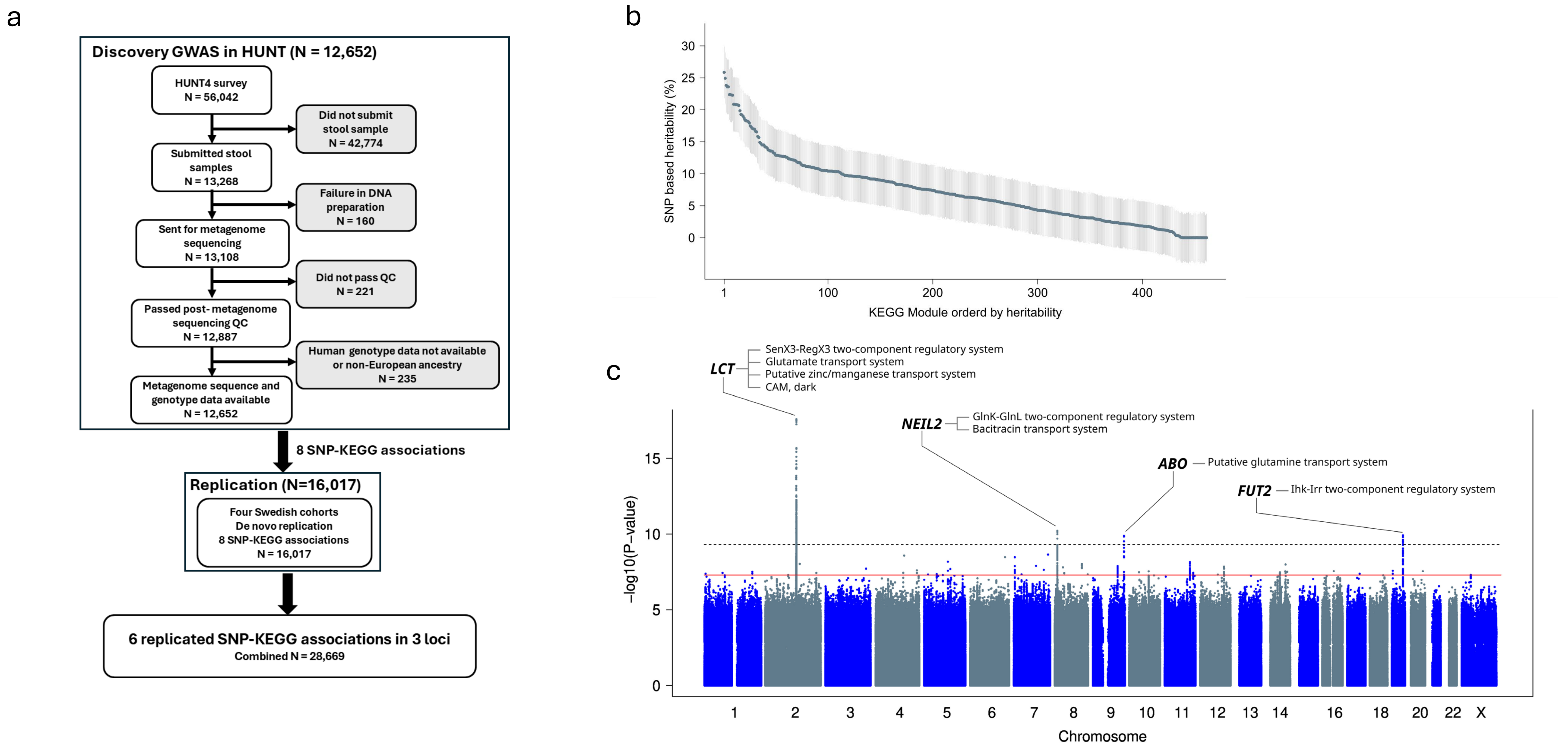

Supplementary Fig. 4 Genome-wide association analysis and replications of KEGG functionality modules. a) Overall design of the discovery GWAS in HUNT and the subsequent replications in four Swedish cohorts. b) Total SNP-based heritability, estimated as the ratio of genetic variance (Vg) to phenotypic variance (Vp), estimated as the ratio of genetic variance (Vg) to phenotypic variance (Vp), with 95 % confidence intervals for the 461 evaluated KEGG functionality modules using genome-wide complex trait analysis (GCTA). c) Manhattan plot summarizing the results of the 461 KEGG functionality modules evaluated in the discovery GWAS. Results after replication are shown in Table 2. The unadjusted p-values are based on two-sided z-tests. Red line = genome-wide significance ($P < 5.0 \times 10^{-8}$). Blue dotted line = study-wide significance threshold adjusted for the number of effective tests ($P < 4.9 \times 10^{-10}$). The identified genetic loci (index SNP ± 500 kbp) are given together with the KEGG functionality modules with associations passing the genome-wide significant threshold.

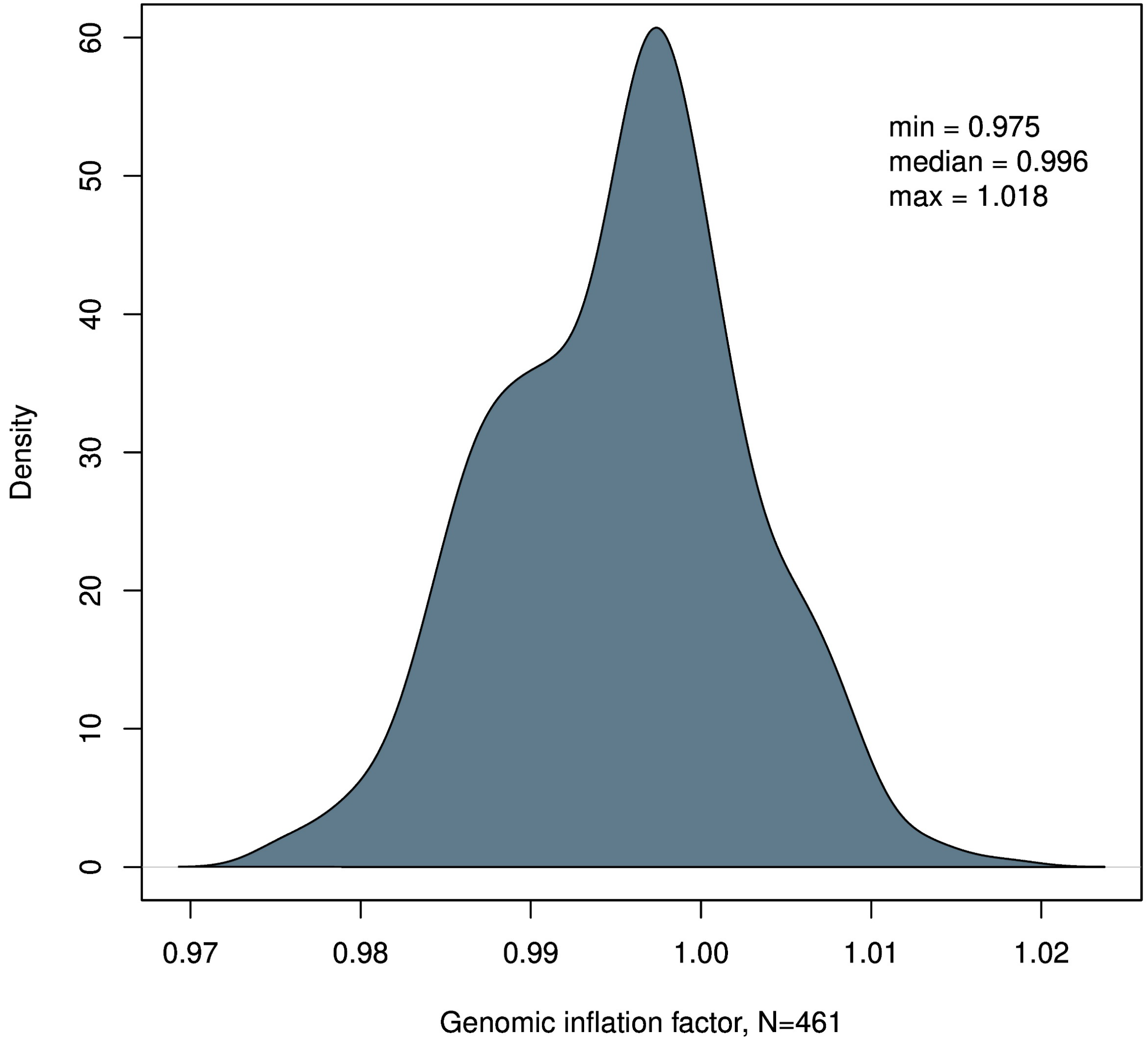

Supplementary Fig. 5 Density plot of the genomic inflation factor for the 461 evaluated gut microbiota KEGG functionality modules.

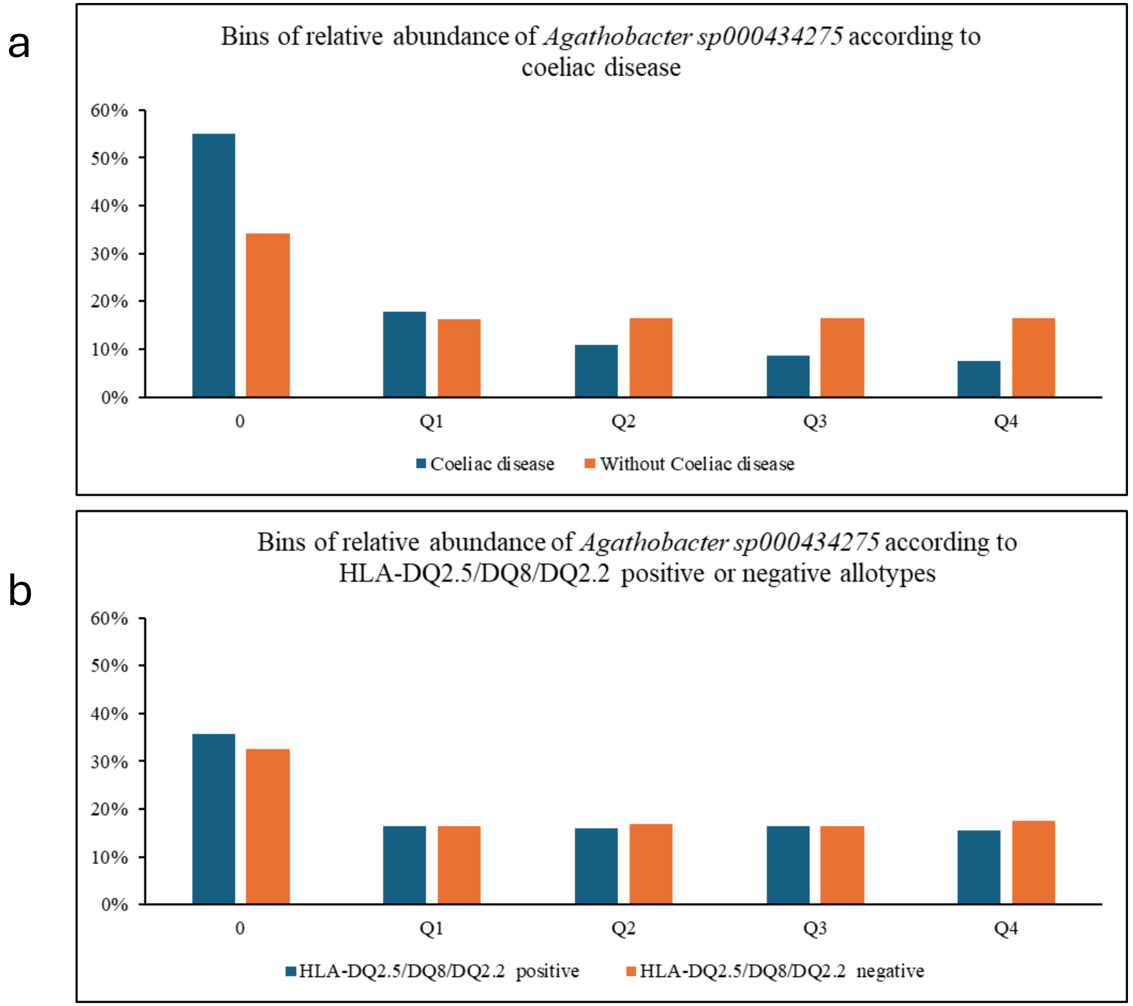

Supplementary Fig. 6 *Agathobactersp000434275* according to coeliac disease and HLA-DQ2.5, HLA-DQ8 and HLA-DQ2.2 in HUNT.

a) Distribution plot of *Agathobactersp000434275* in different bins according to coeliac disease status (N = 240 with and 12,437 without coeliac disease). In the first bin, participants with absence of *Agathobactersp000434275* were included (0) while the remaining participants were divided into four quartiles (Q1 with the lowest levels to Q4 with the highest levels). The proportion with absence of *Agathobactersp000434275* was higher in participants with coeliac disease (55.0 %) compared with those without coeliac disease (33.4 %, $P = 3.8 \times 10^{-11}$, Chi-2 test). b) Distribution plot of *Agathobactersp000434275* in different bins according to the coeliac disease risk allotypes HLA-DQ2.5, HLA-DQ8 and HLA-DQ2.2 (N = 7,105 positive for risk allotypes and 5,529 negative). In the first bin, participants with absence of *Agathobactersp000434275* were included (0) while the remaining participants were divided into four quartiles (Q1 with the lowest levels to Q4 with the highest levels). The proportion with absence of *Agathobactersp000434275* was modestly higher in participants with the coeliac disease risk allotypes HLA-DQ2.5, HLA-DQ8 and HLA-DQ2.2 .5/DQ8DQ2.2 (35.7 %) compared with those without (32.6 %, $P = 2.8. \times 10^{-4}$, Chi-2 test).

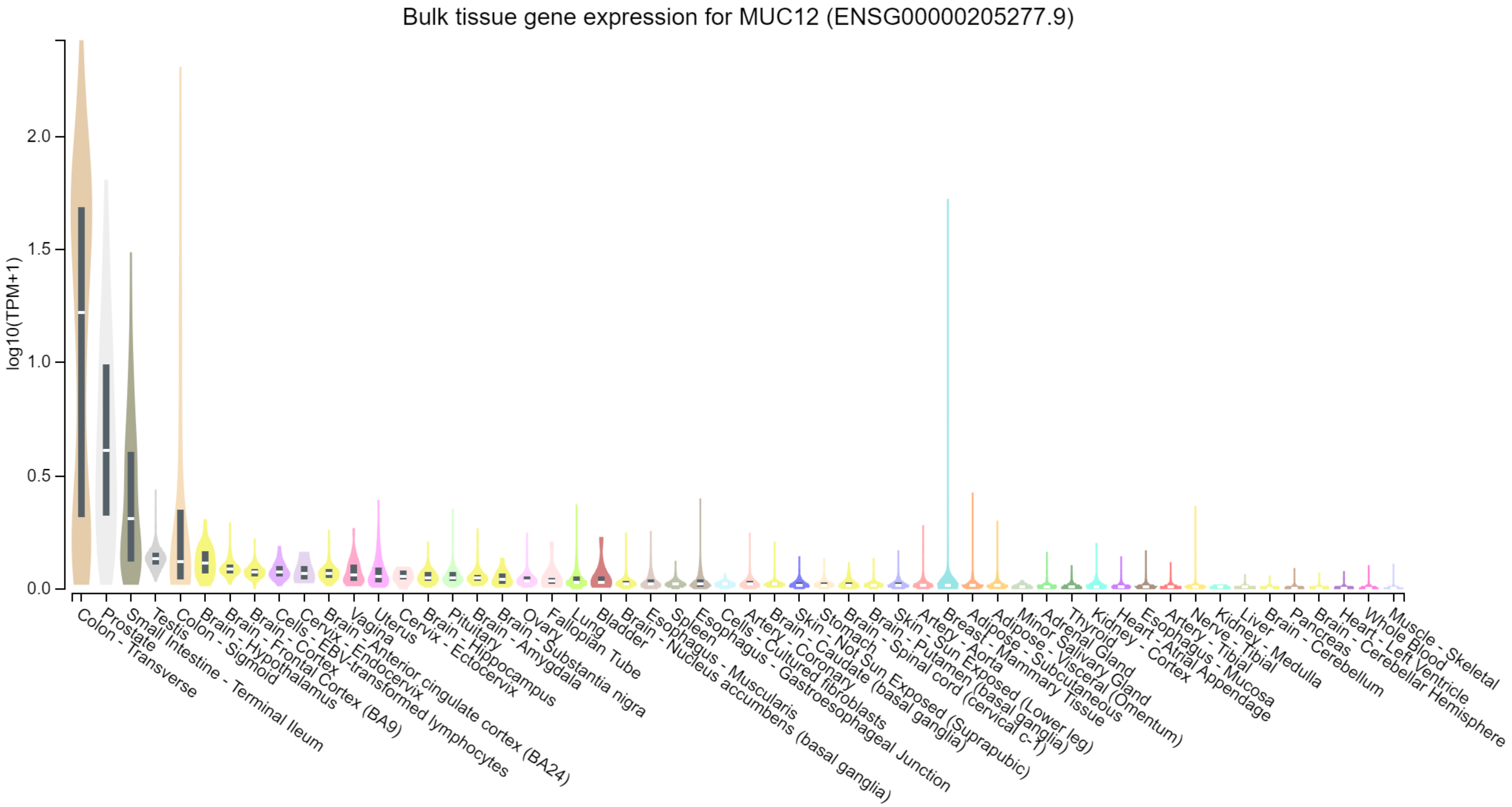

Supplementary Fig. 7 High *MUC12* expression in colon when evaluated among multiple human tissues in the GTEx portal. (https://www.gtexportal.org/home/gene/MUC12).

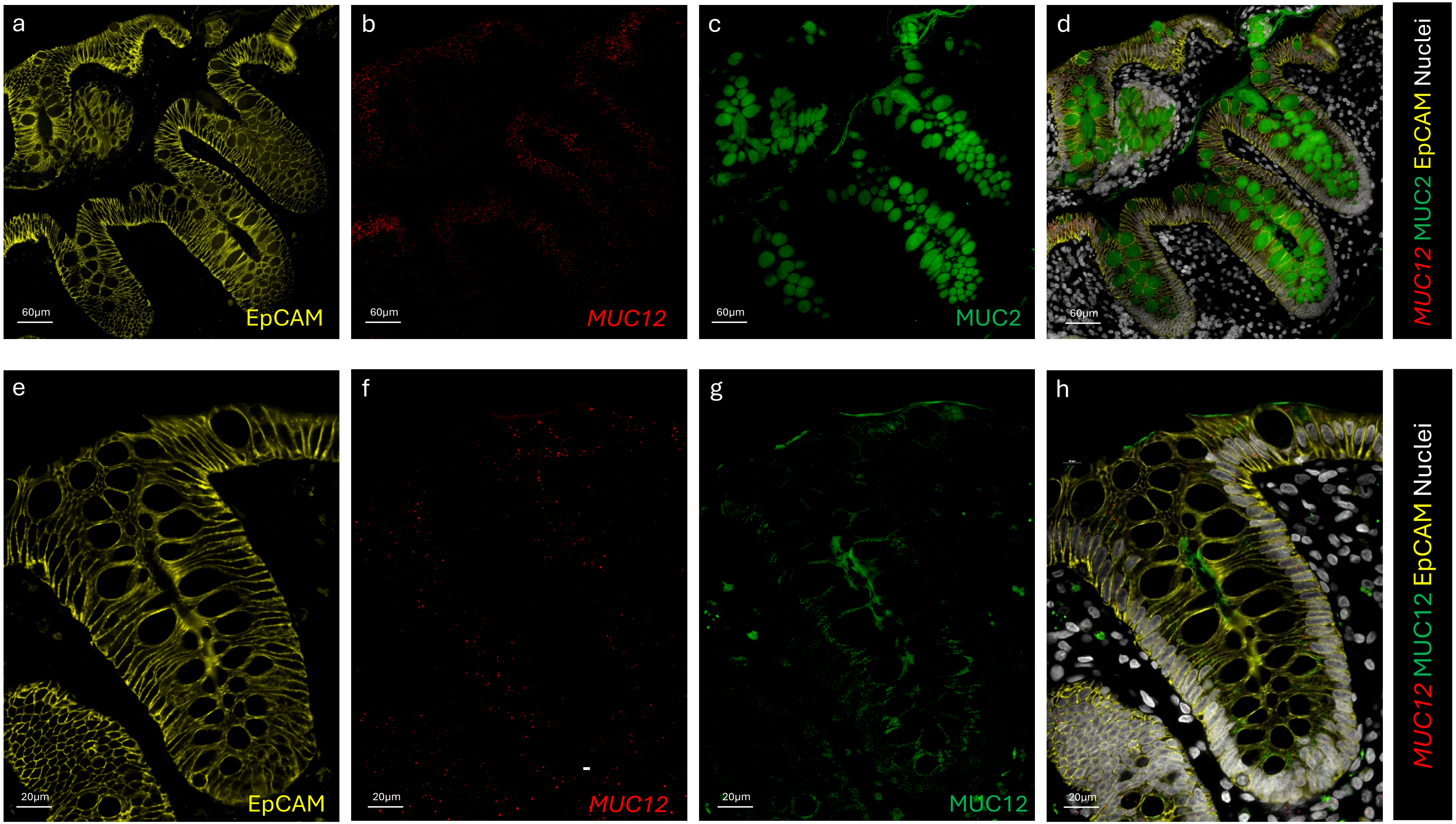

Supplementary Fig. 8 Dual RNAscope and immunohistochemistry of MUC12 in the colon.

a-h) Each stain from the dual fluorescent in situ hybridization (FISH) and immunofluorescence in human sigmoid colon is split into single channels for display. a and e), EpCAM protein marked in yellow. b and f) *MUC12* mRNA in red. c) MUC2 protein in green. g) MUC12 protein in green. d) Combined images of *MUC12* mRNA in red, MUC2 protein as a marker of Goblet cells in green, EpCAM (Epithelial cell adhesion molecule) protein in yellow as a marker of colonocytes and nuclei are indicated in white. h) Combined images of *MUC12* mRNA in red, MUC12 protein in green, EpCAM protein in yellow as a marker of colonocytes and nuclei are indicated in white. The experiment was repeated three times using samples from four different subjects.
