# Supplementary material for: The HUNT study identifies host genetic factors reproducibly associated with human gut microbiota composition
Source: Nat Genet. 2026 Feb 13;58(3):530–9. doi: 10.1038/s41588-026-02502-4 (PMC12987729; doi:10.1038/s41588-026-02502-4)
Supplement: Supplementary file 2 — Reporting Summary [file 41588_2026_2502_MOESM2_ESM.pdf]

nature portfolio

Corresponding author(s): Claes Ohlsson and Marta Riise Moksnes

Last updated by author(s): Sep 5, 2025

Reporting Summary

Nature Portfolio wishes to improve the reproducibility of the work that we publish. This form provides structure for consistency and transparency in reporting. For further information on Nature Portfolio policies, see our Editorial Policies and the Editorial Policy Checklist.

Statistics

For all statistical analyses, confirm that the following items are present in the figure legend, table legend, main text, or Methods section.

n/a	Confirmed	
☐	☒	The exact sample size (*n*) for each experimental group/condition, given as a discrete number and unit of measurement
☒	☐	A statement on whether measurements were taken from distinct samples or whether the same sample was measured repeatedly
☐	☒	The statistical test(s) used AND whether they are one- or two-sided<br>*Only common tests should be described solely by name; describe more complex techniques in the Methods section.*
☐	☒	A description of all covariates tested
☐	☒	A description of any assumptions or corrections, such as tests of normality and adjustment for multiple comparisons
☐	☒	A full description of the statistical parameters including central tendency (e.g. means) or other basic estimates (e.g. regression coefficient) AND variation (e.g. standard deviation) or associated estimates of uncertainty (e.g. confidence intervals)
☐	☒	For null hypothesis testing, the test statistic (e.g. *F*, *t*, *r*) with confidence intervals, effect sizes, degrees of freedom and *P* value noted<br>*Give P values as exact values whenever suitable.*
☒	☐	For Bayesian analysis, information on the choice of priors and Markov chain Monte Carlo settings
☒	☐	For hierarchical and complex designs, identification of the appropriate level for tests and full reporting of outcomes
☐	☒	Estimates of effect sizes (e.g. Cohen's *d*, Pearson's *r*), indicating how they were calculated

Our web collection on statistics for biologists contains articles on many of the points above.

Software and code

Policy information about availability of computer code

Data collection	sequencing at 2 × 150 bp on an Illumina NovaSeq 6000.<br>Gut microbiome profiling was performed using the Clinical Microbiomics Human Microbiome Profiler (CHAMPTM) pipeline which utilizes the Genome Taxonomy Database r214 for taxonomic annotation of prokaryotes. MAGs were clustered by species using the Genome Taxonomy Database Toolkit (GTDB-Tk release R214), while unannotated MAGs were clustered at 95 % identity using FastANI. The catalogue included 6809 microorganisms. PheWAS was performed using the Open Targets platform (PMID: 36399499, May 5 2024).

Data analysis

Most analyses have been performed using R v4.1.1 or higher (https://cran.r-project.org/)
R package vegan v. 2.6-4 was used for alpha diversity estimations
R packages MendelianRandomization was used for Mendelian randomization analyses
R package coloc was used for colocalization analyses

REGENIE v.3.4.1. was used for GWAS (https://github.com/rgcgithub/regenie/releases)
METAL v. 2011-03-25 was used for meta-analyses (https://genome.sph.umich.edu/wiki/METAL)
GCTA v. 1.94.1 was used for heritability estimates
LDSC v1.0.1 was used for heritability estimates (https://github.com/bulik/ldsc)
KING v.2.3.2 was used to determine relationship (https://www.kingrelatedness.com/)

For manuscripts utilizing custom algorithms or software that are central to the research but not yet described in published literature, software must be made available to editors and reviewers. We strongly encourage code deposition in a community repository (e.g. GitHub). See the Nature Portfolio guidelines for submitting code & software for further information.

Data

Policy information about availability of data

All manuscripts must include a data availability statement. This statement should provide the following information, where applicable:

- Accession codes, unique identifiers, or web links for publicly available datasets
- A description of any restrictions on data availability
- For clinical datasets or third party data, please ensure that the statement adheres to our policy

Individual level data from HUNT can be accessed by, or in collaboration with, a Norwegian principal investigator. Researchers can apply for HUNT data access from HUNT Research Centre (https://www.ntnu.edu/hunt) if they have obtained project approval from the Regional Committee for Medical and Health Research Ethics (REC). Information on the application and conditions for data access is available at https://www.ntnu.edu/hunt/data.

For the replication cohorts: The genetic data used in the SCAPIS, SIMPLER, and MOS board, are not shared publicly due to confidentiality. Data will be shared upon reasonable request after permission from the Swedish Ethical Review Authority (https://etikprovningsmyndigheten.se) and from the respective cohort boards (https://www.scapis.org/data-access, https://www.simpler4health.se, and https://www.malmo-kohorter.lu.se/malmo-offspring-study-mos).

Summary statistics of the discovery GWAS are available at the GWAS Catalog under study accession numbers GCST90666541-GCST90667549 (https://www.ebi.ac.uk/gwas).

Genome Taxonomy Database Toolkit (GTDB-Tk release R214) can be found at https://gtdb.ecogenomic.org.

Research involving human participants, their data, or biological material

Policy information about studies with human participants or human data. See also policy information about sex, gender (identity/presentation), and sexual orientation and race, ethnicity and racism.

Reporting on sex and gender

Reporting on sex in all cohorts: Table S2
In HUNT sex is determined by genetics.

Reporting on race, ethnicity, or other socially relevant groupings

The discovery and replication cohorts mainly included participants of European ancestry living in Nordic countries. In the discovery cohort (HUNT), participants that were not of European ancestry or lacked genotype data were excluded, see Fig 1A and Fig S4A.

Population characteristics

Reported in Table S2
The HUNT study is a longitudinal population-based health study conducted in the county of Trøndelag, Norway. Data and biological samples have been collected through four cross-sectional surveys, from 1984-2019 (HUNT1-4). Among 56,042 participants in the HUNT4 survey (2017-2019) 13,268 participants submitted stool samples for gut microbiome profiling and data from 12,887 of these participants passed the post-metagenome sequencing quality control (Fig 1A). A total of 12,652 HUNT4 participants of European ancestry had both genetic and gut microbiome data available and were included in the present GWAS (Fig 1A).

61% of the subjects were women and 39% men. Mean age was 59.9 years (SD 14.1) years. Mean body mass index was 27.1 (SD 4.5) kg/m2. Participants with coeliac disease were identified through serological screening and linkage to hospital journal records and the Norwegian Patient Registry. 240 coeliac participants with available fecal samples from HUNT4 were included in the present study. No treatment was performed. Participants were genotyped using Illumina HumanCoreExome arrays and genotype data were imputed to the Human Reference Consortium (HRC) 1.1 panel and was for this study available for 12.652 participants also having meta-genomesequecincing data available.

Further details in Methods section.

Recruitment

HUNT: All people living in the county of Trøndelag, Norway, that would pass the age of 20 during the period the field stations were in their municipality were eligible to participate in HUNT. Eligible participants, identified through the Norwegian

National Population Register, were invited to HUNT by the HUNT research centre, and if needed, they were reminded once. Among 56,042 participants in the HUNT4 survey (2017-2019) 13,268 participants submitted stool samples for gut microbiome profiling and data from 12,887 of these participants passed the post-metagenome sequencing quality control (Fig 1A). A total of 12,652 HUNT4 participants of European ancestry had both genetic and gut microbiome data available and were included in the present GWAS (Fig 1A).

Among the subjects invited to the HUNT4 visit, there might be a bias of those that decided to participate in HUNT4 (54%). In addition, among the participants in HUNT4, there might be a bias of those who agreed to deliver stool samples (23%). Further details in Methods section.

Ethics oversight

Ethics oversight
HUNT: The local ethical review board approved the study (Regional committee for medical and health research ethics, Midt-Norge; REK-656785) and all participants provided written informed consent.

Replications cohorts: The association analyses performed in the Swedish cohorts have been approved by the Swedish Ethical Review Authority (DNR 2022-06137-01 and DNR 2024-01992-02). Ethical approval and written informed consent were obtained for the individual Swedish cohorts. Ethical approval for SCAPIS was granted by the Swedish Ethical Review Board (DNR 2010-228-31M), and all participants gave written informed consent. The SIMPLER studies received approval from the Swedish Ethical Review Board (DNR 2009/2066-32, DNR 2009/1935-32, DNR 2010/0148-32, DNR 2014/892-31/3), and all participants gave written informed consent. The MOS study received approval from the Ethics Review Committee of Lund University (DNR 2012-594), and all participants gave written informed consent.

Histology study: Was approved by the Research Ethical Committee in Gothenburg, Sweden (Ethical permission 2020-03196)

Note that full information on the approval of the study protocol must also be provided in the manuscript.

Field-specific reporting

Please select the one below that is the best fit for your research. If you are not sure, read the appropriate sections before making your selection.

☒ Life sciences ☐ Behavioural & social sciences ☐ Ecological, evolutionary & environmental sciences

For a reference copy of the document with all sections, see nature.com/documents/nr-reporting-summary-flat.pdf

Life sciences study design

All studies must disclose on these points even when the disclosure is negative.

Sample size

Sample sizes for the cohorts were chosen on the basis of all data available at the time of the analyses.
Sample size HUNT: 12,652. The sample size was not predetermined by sample size calculations. At the time of the start of this study, the used sample size was the largest single cohort ever for GWAS on metagenome-sequence data of gut microbiota taxonomy and and gut microbiota functionality. Thus there was a good chance for us to identify novel genetic signals for gut microbiota composition and/or functionality compared with previous studies.

Replication data set, all available data was used (N=16,040-21,999 dependning on SNP-species association replicated). We used all available feasible replication data sets. This to get reliable replication results.

Further information in Table S2.

Data exclusions

Exclusion of participants is shown in Figure 1A and S2A, stating how many that did not submit stool samples, how many samples of DNA that failed in preparation, and how many samples did not pass QC, and how many that did not have information on genetics.

Genotyping and imputation: Samples were excluded if they had call rate < 99%, large chromosomal copy number variants, contamination > 2.5% as estimated with BAF Regress, or a discrepancy between genetically inferred sex and reported gender. Genetic variants were excluded if they deviated from Hardy-Weinberg Equilibrium (p-value < 10-4). We included genetic variants with minor allele frequency > 0.01 in the analyses.

Replication

We tested 13 SNP-species associations for replication, and 12 of these replicated in the replication data set.
We tested 8 SNP-KEGG associations for replication and 6 of these replicated in the replication data set.

Randomization

Not applicable (GWAS study, not a randomized trial). Since we did not include a treatment in the present study, there was no rationale for randomization of the participants in the GWAS.

Blinding

Not applicable (GWAS study, not a randomized trial). Since we did not include a treatment in the present study, there was no rationale for blinding of the data in the GWAS.

Reporting for specific materials, systems and methods

We require information from authors about some types of materials, experimental systems and methods used in many studies. Here, indicate whether each material, system or method listed is relevant to your study. If you are not sure if a list item applies to your research, read the appropriate section before selecting a response.

Materials & experimental systems

n/a	Involved in the study
☐	☒ Antibodies
☒	☐ Eukaryotic cell lines
☒	☐ Palaeontology and archaeology
☒	☐ Animals and other organisms
☒	☐ Clinical data
☒	☐ Dual use research of concern
☒	☐ Plants

Methods

n/a	Involved in the study
☒	☐ ChIP-seq
☒	☐ Flow cytometry
☒	☐ MRI-based neuroimaging

Antibodies

Antibodies used	Anti-MUC12-S2 polyclonal rabbit antibody was produced in the lab of Dr. Thaher Pelaseyed at Gothenburg University in Sweden (thaher.pelaseyed@medkem.gu.se).<br>Primary antibodies against either EpCAM (1:250, Abcam, ab71916, lot no 1076051-3) and MUC2-C3 (1:100, GeneTex, GTX100664, lot no 44818) or EpCAM and MUC12-S2 (1:250).<br>Secondary antibodies: Goat anti-Mouse IgG, IgM (H+L) Secondary Antibody, Alexa Fluor™ 488 (ThermoFisher, A10680, lot no 1664758, diluted 1:400) or Cy™3 AffiniPure® Donkey Anti-Rabbit IgG (H+L) (Jackson ImmunoResearch, catalog no. 711-165-152, lot no 171768, diluted 1:400)
Validation	AntiEpCAM antibody: validated for human samples fixed in formalin and embedded in paraffin; www.abcam.com/en-se/products/primary-antibodies/epcam-antibody-ab71916#<br>MUC2-C3 antibody: validated for human samples embedded in paraffin; www.genetex.com/Product/Detail/MUC2-antibody-C3-C-term/GTX100664?srsltid=AfmBOop-0egDEmgF6dRR3SOQzY1SIxPp7p1ZSmlFnYi5S5LoyLyoFDtU<br>Anti-MUC12-S2 antibody: produced by the lab of Dr. Thaher Pelaseyed (see methods) and the specificity of the anti-MUC12-S2 polyclonal antibody was validated via peptide block on tissue sections from the human colon.

Plants

Seed stocks	NA
Novel plant genotypes	NA
Authentication	NA
